# Clinical and cost-effectiveness of a diabetes education and behavioural weight management programme versus a diabetes education programme in adults with a recent diagnosis of type 2 diabetes: study protocol for the Glucose Lowering through Weight management (GLoW) randomised controlled trial

**DOI:** 10.1136/bmjopen-2019-035020

**Published:** 2020-04-28

**Authors:** Amy L Ahern, Jenny Woolston, Emma Wells, Stephen J Sharp, Nazrul Islam, Emma Ruth Lawlor, Robbie Duschinsky, Andrew J Hill, Brett Doble, Ed Wilson, Stephen Morris, Carly A Hughes, Alan Brennan, Jennifer Bostock, Clare Boothby, Simon J Griffin

**Affiliations:** 1 MRC Epidemiology Unit, University of Cambridge School of Clinical Medicine, Cambridge, Cambridgeshire, UK; 2 Primary Care Unit, Institute of Public Health, University of Cambridge School of Clinical Medicine, Cambridge, Cambridgeshire, UK; 3 School of Medicine, University of Leeds, Leeds, West Yorkshire, UK; 4 Programme in Health Services and Systems Research, Duke-NUS Medical School, Singapore; 5 Norwich Medical School, University of East Anglia, Norwich, UK; 6 Patient and Public Involvement Representative, Fakenham Medical Practice, Fakenham, Norfolk, UK; 7 Faculty of Medicine and Health Sciences, University of East Anglia, Norwich, Norfolk, UK; 8 School of Health and Related Research, The University of Sheffield, Sheffield, UK; 9 Patient and Public Involvement Representative, Kent, UK

**Keywords:** weight management, type 2 diabetes, diabetes education, primary care, randomised controlled trial

## Abstract

**Introduction:**

People with type 2 diabetes (T2D) can improve glycaemic control or even achieve remission through weight loss and reduce their use of medication and risk of cardiovascular disease. The Glucose Lowering through Weight management (GLoW) trial will evaluate whether a tailored diabetes education and behavioural weight management programme (DEW) is more effective and cost-effective than a diabetes education (DE) programme in helping people with overweight or obesity and a recent diagnosis of T2D to lower their blood glucose, lose weight and improve other markers of cardiovascular risk.

**Methods and analysis:**

This study is a pragmatic, randomised, single-blind, parallel group, two-arm, superiority trial. We will recruit 576 adults with body mass index>25 kg/m^2^ and diagnosis of T2D in the past 3 years and randomise them to a tailored DEW or a DE programme. Participants will attend measurement appointments at a local general practitioner practice or research centre at baseline, 6 and 12 months. The primary outcome is 12-month change in glycated haemoglobin. The effect of the intervention on the primary outcome will be estimated and tested using a linear regression model (analysis of covariance) including randomisation group and adjusted for baseline value of the outcome and the randomisation stratifiers. Participants will be included in the group to which they were randomised, under the intention-to-treat principle. Secondary outcomes include 6-month and 12-month changes in body weight, body fat percentage, systolic and diastolic blood pressure and lipid profile; probability of achieving good glycaemic control; probability of achieving remission from diabetes; probability of losing 5% and 10% body weight and modelled cardiovascular risk (UKPDS). An intention-to-treat within-trial cost-effectiveness analysis will be conducted from NHS and societal perspectives using participant-level data. Qualitative interviews will be conducted with participants to understand why and how the programme achieved its results and how participants manage their weight after the programme ends.

**Ethics and dissemination:**

Ethical approval was received from East of Scotland Research Ethics Service on 15 May 2018 (18/ES/0048). This protocol (V.3) was approved on 19 June 2019. Findings will be published in peer-reviewed scientific journals and communicated to other stakeholders as appropriate.

**Trial registration number:**

ISRCTN18399564.

**Strengths and limitations of this study**

- This trial will provide robust evidence of the clinical and cost-effectiveness of a scalable tailored diabetes education and behavioural weight management programme versus diabetes education alone in adults with a recent diagnosis of type 2 diabetes.
- If shown to be cost-effective, the intervention being evaluated could be readily integrated into existing UK care pathways and delivered to large numbers of people.
- The behavioural programme is already widely available across many countries and this model of care could be readily adopted across healthcare services internationally.
- This trial only includes follow-up at 6 and 12 months and longer term data may be needed to understand the longer term impact of these programmes.
- Consent has been obtained to follow-up participants through national registries and medical records for up to 15 years.

## Background

The treatment of diabetes and related complications (eg, cardiovascular disease, amputation, kidney failure) uses approximately 10% of the UK NHS budget.^1^ This is predicted to rise to 17% in 2035 as the number of people with diabetes in the UK rises to 6.25 million, of which 5.6 million cases will be adults with type 2 diabetes (T2D).^1^ Adults who are living with T2D are at increased risk of developing physical and mental health comorbidities and have reduced quality of life and shorter life expectancy.^2 3^ There are considerable social and economic costs to the individual living with diabetes as well as to wider society.^1 2 4^

While T2D is typically characterised as a progressive, irreversible condition, there is evidence of remission in patients losing weight through bariatric surgery^5 6^ or closely supervised very-low-calorie formula diets.^7 8^ However, many patients with T2D may be unsuitable for or unwilling to undergo these interventions and, given their high cost and reliance on specialists, they are unlikely to be widely adopted in the NHS in the near future. Partial or complete remission of T2D has also been observed following smaller weight losses achieved through behavioural interventions.^9^ Moreover, even without remission, weight loss and behaviour change can lead to improvements in health outcomes in people who have diabetes. We have shown that losing a moderate amount of weight or making healthy behaviour changes (eg, increasing physical activity, reducing alcohol, energy and fat intake) in the first year after diagnosis can reduce the likelihood of stroke or heart attack in the next 5–10 years^10–12^ and increase the likelihood of achieving remission at 5 years.^13^

The Look AHEAD trial demonstrated that intensive specialist-led behavioural programmes could lead to weight loss and reductions in cardiovascular risk factors over 8-year follow-up.^14^ However, there are currently insufficient resources in the UK NHS to provide intensive, specialist-led behavioural programmes to 3.2 million individuals who have T2D and the additional 200 000 individuals who are diagnosed each year. Instead, current guidelines focus on structured diabetes education (DE) and dietary advice,^15^ which is cheaper and scalable but has small, short-term effects on weight and glycaemia, and relatively poor uptake.^16–18^ A recent systematic review found that supportive behaviour change programmes (with ≥11 hours of contact time) achieve greater reductions in weight and glycated haemoglobin (HbA_1c_) than structured education without additional support (≤10 hours).^17^ Integrating effective but scalable behaviour change programmes into care pathways for T2D could potentially improve patients’ glycaemic control and related risk factors and reduce complications. This would improve health and quality of life for people living with diabetes and reduce the burden of diabetes on healthcare resources.

We have previously shown that commercial open-group behavioural weight management programmes, such as WW (formerly Weight Watchers) or Slimming World, offer a scalable and cost-effective way to help people lose weight and reduce risk of diabetes.^19–22^ A randomised trial in the USA showed that a combination of WW classes and remote dietary counselling achieved greater weight losses and reductions in HbA_1c_ than standard care over 1 year in people with diabetes.^23^ A quarter of participants randomised to this programme achieved good glycaemic control (HbA_1c_ below 53 mmol/mol) at 12 months compared with 14% of those receiving standard care. In the UK, a similar intervention has been developed for use in the NHS that combines referral to WW with NICE-compliant DE and dietary advice. However, this programme is unlikely to be widely commissioned without robust evidence of cost-effectiveness. The proposed trial will provide reliable evidence on the relative effectiveness and cost-effectiveness of a tailored diabetes education and behavioural weight management programme (DEW) versus DE, for people with a recent diagnosis of T2D (≤3 years).

## Objectives

### Primary objective

To evaluate the effect of tailored DEW versus DE on HbA_1c_ at 12 months in adults with a recent diagnosis of T2D.

### Secondary objectives

To evaluate the effect of DEW versus DE on:

- body weight, body fat percentage, blood pressure, lipid profile and modelled 10-year cardiovascular risk at 6 and 12 months
- probability of achieving clinically significant weight loss, good glycaemic control or diabetes remission at 6 and 12 months
- changes in diet and physical activity at 6 and 12 months
- psychosocial factors associated with successful weight control at 6 and 12 months.

To evaluate the within-trial cost-effectiveness of DEW vs DE.

To assess the extent to which the two programmes reach the target population.

To explore participant and practitioner experiences of the two programmes and the extent to which these programmes meet their needs.

To clarify causal mechanisms and identify contextual factors associated with variations in outcome.

To identify barriers and facilitators to maintenance of weight management behaviours after treatment cessation.

## Methods and analysis

### Trial design

**Figure 1:**
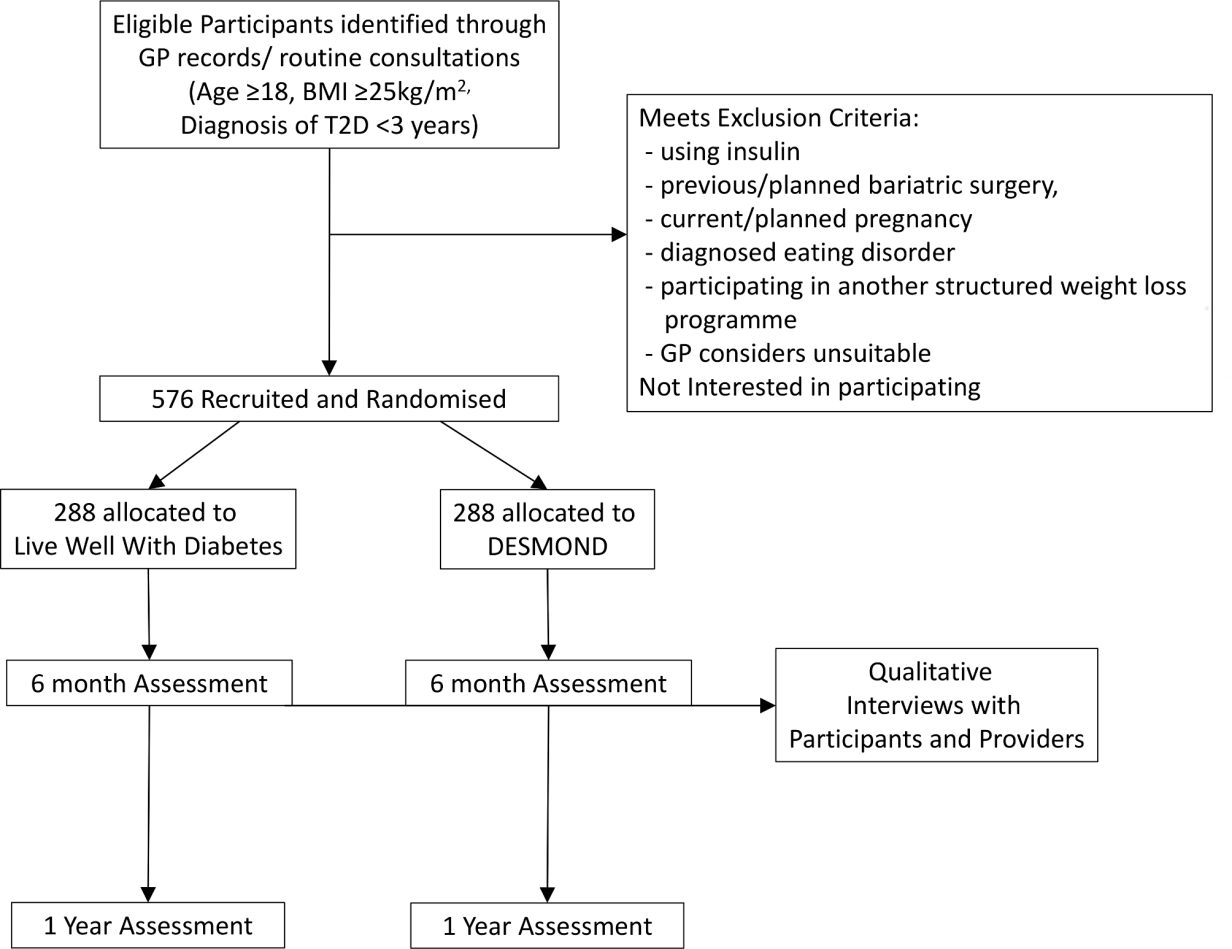
Participant flow diagram.

This is a pragmatic, randomised, single-blind, parallel group, two-arm, superiority trial. Participants will be randomised to either the tailored DEW or to DE. Block randomisation will be used with a 1:1 allocation stratified by sex and duration of diabetes (figure 1).

### Study setting

Research active primary care practices which currently refer patients with T2D (≤3 years duration) to local structured DE (Diabetes and Education Self-Monitoring for Ongoing and Newly Diagnosed (DESMOND)) and have active WW groups in the local community.

### Participants

Participants will be 576 adults with overweight or obesity who have a diagnosis of T2D in the past 3 years.

#### Inclusion criteria

- Body mass index≥25 kg/m^2^.
- Age≥18 years.
- Diagnosis of T2D within the previous 36 months (confirmatory blood test will not be required).
- Capable of giving informed consent.
- Have a good understanding of the English language (study materials are not tailored to support non-English language speakers).
- Willing to be randomised.
- Willing to attend follow-up visits at a local participating general practitioner (GP) practice or research centre.

#### Exclusion criteria

- Using insulin.
- Previous/planned bariatric surgery.
- Current/planned pregnancy.
- Current diagnosis of eating disorder.
- Already received a structured DE programme.
- GP considers unsuitable.
- Participation in another structured behaviour change programme for diet and/or physical activity within the past 3 months.

## Interventions

### ​Tailored Diabetes Education and Weight management

The tailored DEW programme was developed and is delivered by WW. It lasts 6 months and is overseen by a registered dietitian with experience in diabetes, diet and behaviour counselling and specific training in the standard WW programme.

#### ​Structured DE

The structured DE programme is delivered remotely (video conferencing/telephone) on a 1:1 basis by the registered dietitian over two education sessions, 3–5 weeks apart (total time 1 hour 30 min, divided between two calls). It covers a standardised QISMET-accredited core curriculum but can then be adapted for the individual’s needs. Additional self-help education materials to support the curriculum will be available online and are delivered to all participants via email or mail at the patient’s preference. These materials may be minimally tailored to the individual participant. Information on the materials can be found at www.weightwatchers.com/uk/live-well-diabetes. During the education sessions, materials on goal setting, understanding diabetes, the glycaemic index, carbohydrates, physical activity and weight management are reviewed.

#### ​Behavioural programme

The behavioural weight management component consists of free membership of WW for 6 months. This includes attendance at weekly in-person group meetings (30–40 min) held at a variety of times in a range of local community venues. These are open-group meetings (new people may join or leave the group at any time) and are led by a coach (trained lay person with experience of the programme). Meetings include a confidential weigh in with the coach and a 30 min interactive education session led by the coach which includes advice on diet, physical activity and positive mindset, using behavioural strategies (eg, goal setting, self-monitoring, problem solving, modifying the personal food environment and relapse prevention). Peer support is available from coaches and other group members. Participants can be accompanied by a friend, relative or carer. Participants will also have access to the WW app, online digital tools and standard materials such as recipe booklets, physical activity guidance and meal trackers for the duration of the intervention. Participants can contact their coach for support/advice between meetings via an online chat function.

#### ​Participant engagement

Once a referral is received, the registered dietitian will telephone the participants, welcome them to the programme, sign them up to a local WW meeting and arrange the first education session. Participants are also given an information sheet with details of how to contact the dietitian directly. A closed social media support group will be formed and all participants will be encouraged to join if they wish to do so. Activity on the group forum will be monitored and supported by the registered dietitian. Each week, the dietitian will contact 2–5 participants (selected at random from those who have participated in the programme for longer than 3 months) via the social media group or phone. Participants can also contact the dietitian proactively during the intervention period for additional remote support where needed, but they will be encouraged to do so via the social media group in the first instance. If participants miss four or more WW standard in-person meetings, their local coach will call to help the individual return to the programme.

### ​Diabetes education

We will recruit from Clinical Commissioning Groups (CCGs) where the commissioned standard care DE is the DESMOND programme.^16 18^ This is a structured DE programme for people with a recent diagnosis of T2D (≤3 years since diagnosis). Participants can attend 6 hours of structured self-management group education, covering: thoughts and feelings about diabetes; understanding diabetes and glucose—what happens in the body; understanding risk factors and complications associated with diabetes; understanding monitoring and medication; how to take control—food choices and physical activity and planning for the future. The structured education is delivered in 1 day or 2 half days by two trained healthcare professionals in local healthcare or community venues. Sessions are delivered in groups of up to 10 participants, and participants can bring a friend or partner with them. The education sessions are supported by specially developed resources.

### Outcomes

#### ​Primary outcome

The 12-month change from baseline in HbA_1c_.

#### ​Secondary outcomes

The 6-month change from baseline in HbA_1c_.

The 6-month and 12-month changes from baseline in body weight, body fat percentage, systolic and diastolic blood pressure, total cholesterol, high-density lipoprotein cholesterol and low-density lipoprotein cholesterol.

Good glycaemic control (HbA_1c_ <53 mmol/mol) at 6 and 12 months.

Remission from diabetes (HbA_1c_ <48 mmol/mol and without glucose-lowering medication for ≥2 months) at 6 and 12 months.

Weight loss ≥5% and ≥10% of initial body weight at 6 and 12 months.

Modelled cardiovascular risk (UKPDS) at 12 months.

#### Behavioural and psychosocial outcomes

The 6-month and 12-month changes from baseline in objectively measured physical activity (accelerometry), self-reported physical activity, objective marker of fruit and vegetable intake (plasma carotenoids) and self-reported dietary intake.

The 6-month and 12-month changes from baseline, adjusted for baseline, in dietary restraint, control over food cravings, emotional eating, self-regulatory skills, social support and diabetes-related quality of life.

#### Health economic outcomes

Detailed micro-costing of DEW and DE.

Health and social care resource use over 12 months (medical notes, registry data, resource use questionnaire).

Self-reported out-of-pocket costs and lost productivity (eg, due to days off work, up to 12 months).

The 12-month quality-adjusted life years (QALYs) based on Health-Related Quality of Life (HRQoL) (EQ-5D-5L)^24 25^ and capability/well-being (ICECAP-A).^26 27^

Total and incremental costs from NHS and societal perspectives; incremental net (monetary) benefit; incremental cost-effectiveness and cost-utility ratios; value of information estimates.

#### ​Uptake and adherence

Number and characteristics of participants who:

- are offered the opportunity to participate in the trial.
- enrol in the trial
- attend the intervention.
- adhere to the intervention (including specific intervention components).

A detailed protocol for process evaluation will also be developed.

### Visits and measurements

#### ​Visit schedule

**Figure 2:**
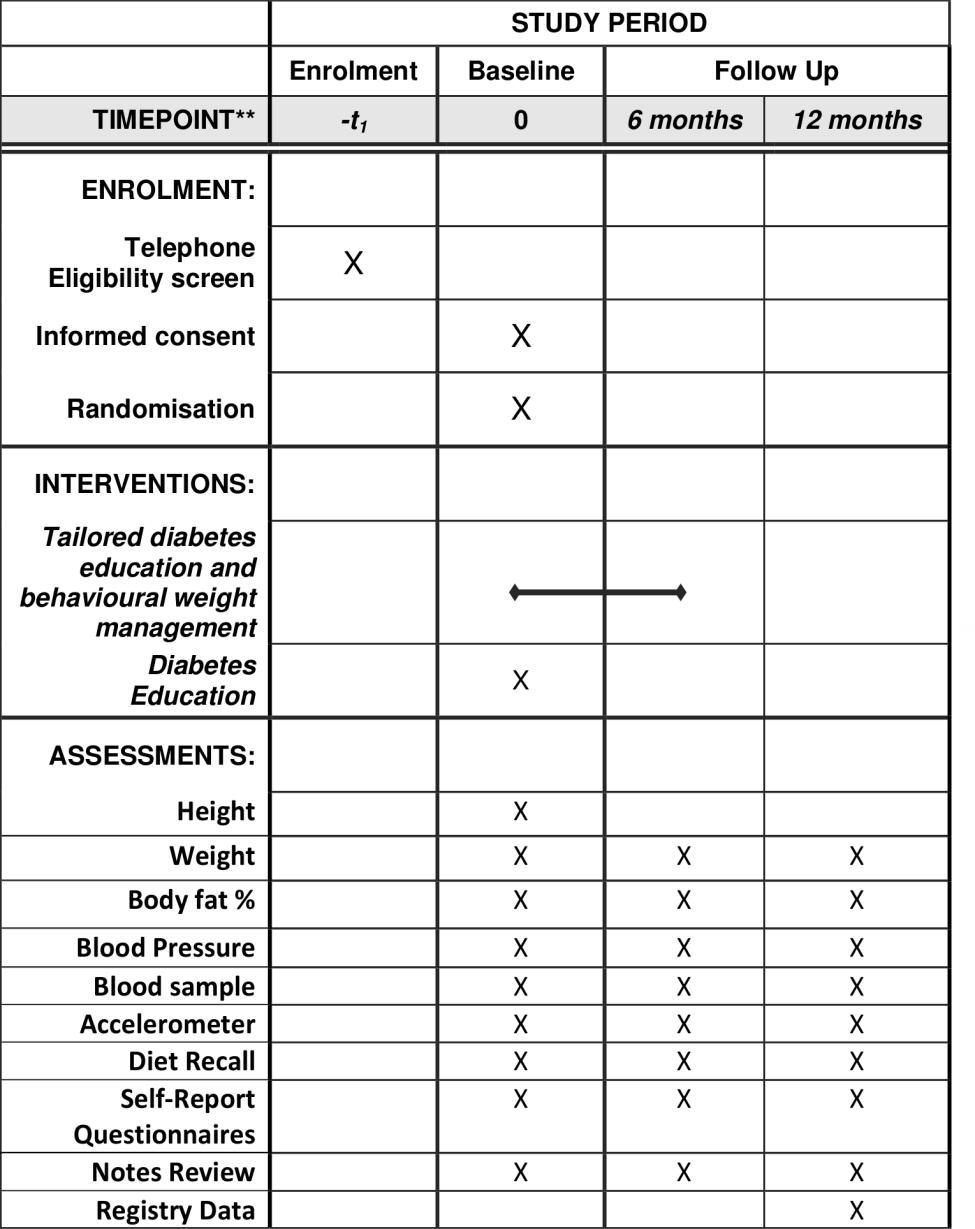
Schedule of enrolment, interventions and assessments.

Participants will be asked to attend measurement appointments at a participating primary care practice or research centre at 0, 6 and 12 months. Details of measures at each assessment are summarised in figure 2. Participants will be reimbursed for reasonable travel expenses and given an honorarium for attending measurement appointments (£10 for baseline and 6-month visits, £30 for the 12-month visit). Honoraria for assessment attendance are not dependent on intervention attendance/completion.

#### ​Anthropometric measures

Anthropometric measurements will be made at participating primary care practices or research centres by research-trained healthcare professionals (hereafter ‘research nurses’) blind to intervention allocation, in line with standardised operating procedures. Participants will be asked to remove shoes and heavy clothing items. Height will be measured in centimetres using a mounted stadiometer (make and model dependent on practice). Where possible, weight (kg) and body fat percentage will be measured using a calibrated Tanita segmental body composition analyser (Tanita Ltd; MA Tokyo, Japan; model dependent on practice) which will be provided to practices by the research team. Where we have an insufficient number of Tanita scales to measure body fat at a practice, we will measure weight (kg) only using calibrated electronic scales (model dependent on practice). To maximise the number of participants for whom we can measure body fat, we will prioritise the use of Tanitas at large practices with a large number of eligible patients, and Clinical Research Networks (CRN)-led practices (as CRN nurses can transport the Tanita scales to numerous practices). Blood pressure will be measured three times in a resting state using a calibrated OMRON automatic blood pressure monitor (OMRON Healthcare UK, Milton Keynes, UK; model dependent on practice).

If a research nurse is not available to conduct follow-up assessments, assessments may be conducted by trained research centre staff. If participants are unable to attend follow-up appointments at a participating practice, the research team may offer appointments at the local hospital or research centre, or home visits. Participants who are unable or unwilling to attend a visit will be asked to provide a self-measured weight. All other participants will also be asked for a self-measured weight at the time of appointment booking to enable us to quantify the degree of misreporting of self-measured weights.

#### ​Biochemical measures

Participants will be asked to provide a blood sample for the measurement of HbA_1c_, lipid profile and carotenoids. If possible, this sample will be taken at the practice at the same time as the anthropometric measurements. Blood samples will be collected in three tubes labelled with an anonymised barcode (4.9 mL serum tube for lipid profile, 2.6 mL Ethylenediaminetetraacetic acid (EDTA) tube for HbA_1c_, 4.9 mL and lithium heparin (LH) tube for carotenoids). The LH tube is light sensitive, so it will be wrapped in foil to avoid degradation. Samples will be placed in a Royal Mail Safebox and posted via first-class mail to the central laboratory for analysis. Each sample is stable for up to 3 days at room temperature. Plasma samples for analysis of carotenoids will be frozen and analysed in batches.

#### ​Behavioural measures

##### Physical activity

Physical activity will be measured objectively using a triaxial accelerometer (Axivity AX3, Newcastle, UK) which measures raw acceleration (m/s^2^). Participants will be asked to wear the accelerometer on their non-dominant wrist continuously for seven consecutive days and nights. The device is small, light and waterproof and is worn like a watch. It does not need to be taken off while showering, bathing or swimming.

At the baseline visit, the nurse will take the participant through the accelerometer instruction sheet. They will demonstrate to the participant how they should wear the monitor on their wrist. The nurse will also ensure that the timing of the monitor and return is feasible for the participant. The study team will initialise the accelerometer and will post it out to the participant with the instruction sheet. The participant will wear the monitor for 7 days and nights and will complete an activity log. For the 6-month and 12-month visits, accelerometers will be mailed out to participants. At the end of 7 days, the participant will be asked to post the device back to the research centre using prepaid, preaddressed materials. Trained staff will download the data from the accelerometer and check it to ensure the integrity of the data recording. This method has been used in large-scale epidemiological studies including the UK Biobank study in 100 000 individuals.^28^ Data will be analysed using established methods used in UK Biobank. The outcome variables of interest are vector magnitude, a measure of total physical activity and intensity distribution, from which duration of time in different levels of physical activity can be inferred.

Self-reported physical activity will be measured using the Recent Physical Activity Questionnaire, a validated measure of four domains of physical activity (leisure time, occupation, commuting and domestic life) that has been used in a number of intervention studies and large-scale epidemiological studies.^29 30^ This will be administered as part of the self-report questionnaires.

##### Diet

Plasma carotenoids will be measured as an objective marker of fruit and vegetable intake. Self-reported dietary intake will be measured at each time point using a version of the previously validated EPIC Food Frequency Questionnaire^31^ that has been adapted for a 6-month recall period. This will be administered as part of the self-report questionnaires.

#### Self-report questionnaires

Participants will complete a demographics questionnaire at baseline based on Progress-Plus^32^ factors (place of residency, race/ethnicity, occupation, gender/sex, religion, education, socioeconomic status, social capital, age, disability, relationship status, caring responsibilities, car ownership, access to the internet).

Self-reported behavioural and psychosocial measures will be collected via validated self-report questionnaires that can be completed on paper or online, at the participant’s preference. A full list of questionnaires can be found in table 1.

**Table 1:** Questionnaires administered in the GLoW trial

Domain	Measure	Time point
Demographics	Bespoke questionnaire based on PROGRESS-Plus^32^	Baseline
Health-related quality of life	EQ-5D-5L^24 25^	All
Capability/well-being	ICECAP-A^26 27^	All
Diabetes-related quality of life	Audit of Diabetes Dependent Quality of Life^35^	All
Health/social care use	Bespoke resource use questionnaire	All
Food cravings	Control of Eating Questionnaire^36^	All
Dietary restraint	Three Factor Eating Questionnaire—Restraint subscale^37^	All
Binge eating	The Binge Eating Scale^38 39^	All
Dietary intake	Food Frequency Questionnaire^31^	All
Physical activity	Recent Physical Activity Questionnaire^29 30^	All
Intervention adherence	Bespoke questionnaire	6 months

GLoW, Glucose Lowering through Weight management.

#### ​Intervention adherence

Adherence data for the tailored DEW (including attendance at weekly WW meetings and remote education sessions, and use of online tools) will be collected by WW as part of an established system for NHS contracts. Self-reported intervention adherence for both programmes will also be collected via a self-report questionnaire.

#### ​Medical notes review

Healthcare resource use will be obtained for all participants via medical notes review and registry data. Primary care records will be used to extract numbers of visits to the GP and community healthcare workers (defined as any primary or community-based health worker such as nurse and allied health professional contacts noted in the patient’s record) and prescribed medications. Last recorded weight, HbA_1c_ value, smoking status and diabetes status will also be extracted. This will help to minimise missing data from missed assessment appointments.

The notes review will be carried out at each study visit and following the end of the study by a research nurse blind to intervention allocation.

- Baseline visit: notes review to cover the 3 months prior to study start
- 6-month visit: notes review to cover from baseline up to 6 months.
- 12-month visit: notes review to cover from 6 months to 12 months

We will obtain consent to conduct future notes reviews after the study has completed (up to 15 years poststudy) for future studies of long-term outcomes.

#### ​Administrative and registry data

We will obtain consent at baseline for future collection of administrative and registry data on health event and healthcare usage information (up to 15 years poststudy). Outcomes will include hospital episode statistics, cardiovascular events, stroke, myocardial infarction, cancer and death and will be obtained from NHS digital, the Healthcare Quality Improvement Partnership and the National Cancer Registration and Analysis Service. This will allow us to evaluate the longer term impact of these programmes on diabetes-related and weight-related morbidity and mortality.

#### ​Qualitative interviews

Around the time of the 6-month follow-up, we will conduct semi-structured interviews with participants from both intervention arms (n≥26). Participants will be recruited in a 2:1 ratio (DEW:DE), as we are interested in comparing the experience of the two groups, but we expect the group receiving the DEW programme to provide the richest data on the experiences of weight loss and weight loss maintenance. Participants will be purposively sampled (demographic characteristics, programme attendance/adherence, weight change). We will also conduct interviews with treatment providers (n≥12) to understand their experience of delivering the intervention. Interviews will be conducted by a trained research associate and audio recorded.

Questions will focus on exploring:

- How did participants and practitioners experience the programme and its components: (1) education; (2) WW meetings; (3) digital tools and (4) remote dietetic counselling?
- What were the causal mechanisms and contextual factors that were associated with different outcomes?
- What were the needs of participants at the end of the programme?
- What were the facilitators and barriers to maintaining behavioural change?

### Recruitment and enrolment

#### ​Recruitment of practices

Primary care practices will be identified and recruited by the CRN. Participating practices must be currently referring patients with a recent diagnosis of T2D to structured DE (DESMOND) as part of standard practice and should have active WW meetings in the local area. They should also be research active with the capacity to recruit and follow-up participants. The CRN will attempt to recruit practices from diverse areas using known characteristics of the practice population to enable recruitment of a sample broadly representative of the target population (UK adults with overweight and obesity and who have a recent diagnosis of T2D).

#### ​Patient identification

Eligible patients will be identified through electronic searches of primary care records and waiting lists for DE. GPs will write to all potentially eligible patients inviting them to participate. This letter will include a participant information sheet and details of how to contact the study team at the research centre. Participants will also receive opportunistic invitations during routine consultations via an invitation letter and participant information sheet to take home with them. Pop-up alerts on patient records (triggered by diagnosis or attempted referral to DE) will be implemented to facilitate opportunistic recruitment. To boost enrolment, advertisements will also be placed in local pharmacies, news media and other relevant settings.

#### ​Enrolment

Patients willing to participate will be asked to contact the research centre for more information. This can be by telephone, email or reply slip. Research centre staff will answer any questions and conduct a telephone screening. If patients are eligible and willing to participate, the research staff will check that they have received the participant information sheet (sending another if they have not) and arrange a baseline assessment with the research nurse at a local participating practice or research centre.

At the baseline visit, the research nurse will take informed consent. This will include ensuring that participants have read the information sheet and had the opportunity to ask questions. They will also confirm eligibility, including an objective measure of height and weight, before taking the rest of the baseline measurements. At the end of each day, the research nurse will email the study coordinator a list of participants who have formally enrolled on the trial.

### Randomisation

#### ​Randomisation sequence

Participants will be allocated to one of the two intervention arms in a 1:1 allocation using individual-level blocked randomisation stratified by sex (men, women) and duration of diabetes (<1 year, 1–3 years) with a block size of 6. The randomisation sequence will be computer-generated by the trial statistician and the randomisation process implemented by the data manager. The sequence will be unknown to all other personnel, including study coordinators, outcome assessors and investigators.

#### ​Method of implementing the allocation sequence

When participant eligibility has been confirmed at the baseline visit, the practice will inform the study coordinator. The study coordinator will enter the participant’s details into the trial database which will automatically assign an intervention to the participant.

The study coordinator (or member of their team) will write to the participant to inform them of their intervention allocation and will also inform the participant’s GP. The study coordinator or the GP (depending on practice and intervention allocation) will also send a referral form to the intervention provider, giving the details of the participant who has been referred to them.

#### ​Blinding

Given the nature of the intervention, it is not possible to blind participants to their intervention group. GPs will be informed of the intervention allocation and participants will be allowed to discuss the intervention with their GP, as they would outside a trial scenario. However, participants will be asked not to reveal their intervention group to outcome assessors (ie, research nurses taking measurements). The trial statistician and the investigators will be blinded to intervention allocation until the database is locked and the primary analysis is complete.

### Statistics and data analysis

#### ​Sample size calculation

The primary outcome is 12-month change from baseline in HbA_1c_. Based on data from a previous trial in adults with a recent diagnosis of T2D,^33^ we assumed a 16 mmol/mol SD, a 0.8 correlation between baseline and follow-up and 25% attrition. In a US trial of a similar intervention in people with T2D of any duration, a difference of 4 mmol/mol was observed between intervention (−3 mmol/mol) and control (+1 mmol/mol) at 12 months.^23^ We need 576 participants to detect a difference between randomised groups of 3 mmol/mol HbA1c with 90% power at a 5% significance level.

#### ​Statistical analysis plan

A detailed statistical analysis plan will be developed and signed off by the Trial Steering Committee (TSC) prior to analysis. Participants will be analysed in the group to which they were randomised, based on the intention-to-treat principle. The intervention effect, representing the baseline-adjusted difference in change from baseline to 12 months in HbA_1c_ between the intervention and control group, will be estimated using a linear regression model including randomisation group, baseline value of HbA_1c_ (ie, analysis of covariance (ANCOVA)) and the randomisation stratifiers (sex, duration of diabetes). The missing indicator method^34^ will be used to ensure inclusion of participants with a missing baseline value of HbA_1c_. Participants with missing values of HbA_1c_ at 12 months will be excluded (ie, a complete-case analysis). If there are >5% of participants with missing values of HbA_1c_ at 12 months, a sensitivity analysis will be performed using multiple imputation by chained equations; full details of this analysis will be provided in the statistical analysis plan.

Continuous secondary outcomes will be analysed using the method described earlier. Binary secondary outcomes will be analysed using a logistic regression model including randomisation group and the randomisation stratifiers.

For the primary outcome, effect modification by (1) sex, (2) index of multiple deprivation (high, low), (3) educational qualification (below postsecondary, postsecondary and above postsecondary) and (4) duration of diabetes (<1 year; 1–3 years) will be tested using an F-test of the relevant multiplicative interaction parameter in the ANCOVA model. If the p value for a particular interaction is <0.05, then the intervention effect and 95% CI will be estimated within the relevant subgroups.

#### ​Economic evaluation

A detailed health economics analysis plan will be developed and signed off by the TSC prior to analysis. An intention-to-treat within-trial cost-effectiveness analysis will be conducted from both the NHS and the societal perspectives using participant-level data. Benefits will be measured by changes in HbA_1c_ at 12 months for the primary analysis and changes in weight (kg), HRQoL (EQ5D-5L), capability/well-being (ICECAP-A) and QALYs at 12 months for the secondary analyses. Resource use data will be extracted from patient-completed questionnaires and validated with primary care notes review. Unit costs will be extracted from standard NHS cost databases and publications (eg, NHS Reference Costs). Costs and benefits will be left undiscounted as follow-up is only 1 year. If required and appropriate, missing data will be imputed using recognised techniques such as multiple imputation. Descriptive statistics for resource use, total costs, HRQoL and capability/well-being at 1 year as well as incremental cost-effectiveness and cost-utility ratios and incremental net (monetary) benefit measures will be reported. We will undertake deterministic and probabilistic sensitivity analyses, presenting the results of the latter as cost-effectiveness acceptability curves. Value of information analysis will quantify the value of reducing the decision uncertainty which will inform whether further research is worthwhile following completion of this study, and if so on which parameters.

#### ​Qualitative evaluation

Qualitative analyses will explore how the programmes were implemented; participant and provider perceptions of the extent to which the programmes met patient needs and factors participants and providers regarded as causally significant. Analyses will also explore the key challenges anticipated around treatment cessation and weight loss maintenance, and the value attached to the possibility of freedom from or reduction in medication and the concept of ‘remission’.

Recordings will be transcribed by an experienced external agency and checked for accuracy by the research team. Verbatim transcripts will be coded using NVivo software, retaining a focus on narrative sequences and transitions as well as salient themes. A dual coding approach will be used: a first inductive round based on emerging themes relating to the research questions and a second round sensitised by quantitative findings. In the first inductive stage, open codes will be generated based on line-by-line scrutiny of verbatim transcripts uploaded into NVivo. Inconsistencies between coders will be resolved through discussion. Patient and public involvement (PPI) representatives will assist with the analysis of qualitative data; this will include coding a subsample of transcripts following training, and ensuing dialogue.

### Trial Steering Committee

The TSC will provide overall supervision for the GLoW Trial on behalf of the Trial Sponsors (NHS Cambridgeshire and Peterborough CCG, University of Cambridge) and Trial Funder (NIHR Clinical Commissioning Facility) and ensure that the project is conducted to the rigorous standards set out in the UK Policy Framework for Health and Social Care Research and the Guidelines for Good Clinical Practice. The TSC will provide advice to the investigators on all aspects of the trial and will review and agree the trial protocol, the statistical analysis plan and any amendments to the protocol. The TSC will be chaired by Professor Andrew Farmer (University of Oxford). Independent members include Professor Lucy Yardley (University of Southampton), Dr Thomas Fanshawe (University of Oxford), Dr Edel Doherty (NUI Galway), Mr Graham Rhodes (PPI representative) and Ms Hazel Patel (PPI representative). This is a low-risk trial in which participants in both trial groups are referred to accredited education programmes. There are no rules for early stopping and participants and GPs are not blind to intervention allocation. Thus a separate data monitoring committee is not deemed to be necessary.

### Patient and public involvement

PPI informs the design, management, analysis and dissemination of the GLoW study. The initial ideas and research proposal were reviewed by three members of Fakenham Weight Management Service and six members of the University of Cambridge PPI Panel. A PPI representative (JB) is a member of our investigator team and has contributed to the design of the protocol. She will also contribute to designing and delivering PPI training, preparing ethics and R&D submissions, co-authoring journal articles and the final report, disseminating findings to a wide range of audiences and supporting other PPI members. Two PPI representatives are members of the TSC. They will review the final study reports and contribute to the writing of specific sections, such as the lay summary.

To maximise participant engagement and retention and minimise burden, PPI representatives review the content, design and delivery of participant-facing materials. PPI representatives will also support the design of the qualitative interview schedule and the analysis and interpretation of qualitative data. Including PPI perspectives in plans for dissemination will ensure that we access an appropriate range of audiences and communicate messages effectively. PPI representatives will advise on content and methods of dissemination and will review public facing documents such as newsletters and press releases.

PPI representatives will be reimbursed for their time and expenses in a timely manner, and tailored PPI training will be provided to suit the specific needs of the individuals and their role.

### Ethics and dissemination

The MRC Epidemiology Unit has an over-arching data management policy (DMP) that encompasses the standards and processes applied to all research and operational activities in the Unit. A study-specific data management plan, based on this DMP, details how data will be collected, stored, transferred, accessed and archived. The principal investigators (PIs) will ensure that all data generated, stored and shared from this trial will be handled in compliance with the DMP and the General Data Protection Regulations.

All specified analyses will be written up as scientific papers and submitted for publication in peer-reviewed open-access journals. Members of the research team will be involved in reviewing drafts of the manuscripts, abstracts and any other publications arising from the trial. The PIs will have final approval on all publications and any press release, where appropriate. Authorship will be determined using ICMJE criteria. On publication of the main findings, participants will be sent a newsletter that describes the results and gives details of whom to contact to ask questions or obtain further information. Newsletters will be prepared with input from PPI representatives. Where appropriate, we will communicate our findings to local and national stakeholders via tailored summaries of the key findings and by presentations at meetings of local and national networks. Representatives from these groups will be involved in our research throughout and will support us in identifying opportunities for dissemination.

### Trial status

This protocol (V.3) included additional strategies to boost recruitment and was approved on 19 June 2019. Recruitment for the trial began on 18 July 2018 and is expected to close in August 2020.

## Supplementary Material

Reviewer comments

Author's manuscript

## References

[1] Hex N, Bartlett C, Wright D, et al Estimating the current and future costs of type 1 and type 2 diabetes in the UK, including direct health costs and indirect societal and productivity costs. Diabet Med 2012;29:855–62. 10.1111/j.1464-5491.2012.03698.x 22537247

[2] HQIP, NHS, DUK National diabetes audit, 2015–16 report 2a : complications and mDiabetes Audit, 2015–16 Report 2a : Complications and Mortality, 2017.

[3] Ali S, Stone MA, Peters JL, et al The prevalence of co-morbid depression in adults with type 2 diabetes: a systematic review and meta-analysis. Diabet Med 2006;23:1165–73. 10.1111/j.1464-5491.2006.01943.x 17054590

[4] Kanavos P, Den ASV, Schurer W Diabetes expenditure, burden of disease and management in 5 EU countries. LSE Health, 2012: 1–113. http://eprints.lse.ac.uk/54896/1/__libfile_REPOSITORY_Content_LSEHealthandSocialCare_Jan2012_LSEDiabetesReport26Jan2012.pdf

[5] Ribaric G, Buchwald JN, McGlennon TW Diabetes and weight in comparative studies of bariatric surgery vs conventional medical therapy: a systematic review and meta-analysis. Obes Surg 2014;24:437–55. 10.1007/s11695-013-1160-3 24374842 PMC3916703

[6] Sjöström L, Peltonen M, Jacobson P, et al Association of bariatric surgery with long-term remission of type 2 diabetes and with microvascular and macrovascular complications. JAMA 2014;311:2297–304. 10.1001/jama.2014.5988 24915261

[7] Steven S, Hollingsworth KG, Al-Mrabeh A, et al Very low-calorie diet and 6 months of weight stability in type 2 diabetes: pathophysiological changes in responders and nonresponders. Diabetes Care 2016;39:808–15. 10.2337/dc15-1942 27002059

[8] Lean ME, Leslie WS, Barnes AC, et al Primary care-led weight management for remission of type 2 diabetes (direct): an open-label, cluster-randomised trial. Lancet 2018;391:541–51. 10.1016/S0140-6736(17)33102-1 29221645

[9] Gregg EW, Chen H, Wagenknecht LE, et al Association of an intensive lifestyle intervention with remission of type 2 diabetes. JAMA 2012;308:2489–96. 10.1001/jama.2012.67929 23288372 PMC4771522

[10] Long GH, Cooper AJM, Wareham NJ, et al Healthy behavior change and cardiovascular outcomes in newly diagnosed type 2 diabetic patients: a cohort analysis of the ADDITION-Cambridge study. Diabetes Care 2014;37:1712–20. 10.2337/dc13-1731 24658389 PMC4170180

[11] Strelitz J, Ahern AL, Long GH, et al Moderate weight change following diabetes diagnosis and 10 year incidence of cardiovascular disease and mortality. Diabetologia 2019;62:1391–402. 10.1007/s00125-019-4886-1 31062041 PMC6647260

[12] Strelitz J, Ahern AL, Long GH, et al Changes in behaviors after diagnosis of type 2 diabetes and 10-year incidence of cardiovascular disease and mortality. Cardiovasc Diabetol 2019;18:98 10.1186/s12933-019-0902-5 31370851 PMC6670127

[13] Dambha-Miller H, Day AJ, Strelitz J, et al Behaviour change, weight loss and remission of type 2 diabetes: a community-based prospective cohort study. Diabet Med 2019 10.1111/dme.14122. [Epub ahead of print: 03 Sep 2019]. PMC7155116 31479535

[14] Look AHEAD Research Group Eight-Year weight losses with an intensive lifestyle intervention: the look ahead study. Obesity 2014;22:5–13. 10.1002/oby.20662 24307184 PMC3904491

[15] National Institute for Health and Care Excellence NICE NG28. type 2 diabetes in adults: management, 2015: 1–86. http://www.nice.org.uk/guidance/ng28/resources/type-2-diabetes-in-adults-management-1837338615493 26741015

[16] Davies MJ, Heller S, Skinner TC, et al Effectiveness of the diabetes education and self management for ongoing and newly diagnosed (DESMOND) programme for people with newly diagnosed type 2 diabetes: cluster randomised controlled trial. BMJ 2008;336:491–5. 10.1136/bmj.39474.922025.BE 18276664 PMC2258400

[17] Pillay J, Armstrong MJ, Butalia S, et al Behavioral programs for type 2 diabetes mellitus: a systematic review and network meta-analysis. Ann Intern Med 2015;163:848–60. 10.7326/M15-1400 26414227

[18] Gillett M, Dallosso HM, Dixon S, et al Delivering the diabetes education and self management for ongoing and newly diagnosed (DESMOND) programme for people with newly diagnosed type 2 diabetes: cost effectiveness analysis. BMJ 2010;341:c4093 10.1136/bmj.c4093 20729270 PMC2924963

[19] Ahern AL, Wheeler GM, Aveyard P, et al Extended and standard duration weight-loss programme referrals for adults in primary care (wrap): a randomised controlled trial. Lancet 2017;389:2214–25. 10.1016/S0140-6736(17)30647-5 28478041 PMC5459752

[20] Meads DM, Hulme CT, Hall P, et al The cost-effectiveness of primary care referral to a UK commercial weight loss programme. Clin Obes 2014;4:324–32. 10.1111/cob.12077 25826162

[21] Fuller NR, Colagiuri S, Schofield D, et al A within-trial cost-effectiveness analysis of primary care referral to a commercial provider for weight loss treatment, relative to standard care—an international randomised controlled trial. Int J Obes 2013;37:828–34. 10.1038/ijo.2012.139 PMC3679478 22929209

[22] Jebb SA, Ahern AL, Olson AD, et al Primary care referral to a commercial provider for weight loss treatment versus standard care: a randomised controlled trial. Lancet 2011;378:1485–92. 10.1016/S0140-6736(11)61344-5 21906798 PMC3207352

[23] O'Neil PM, Miller-Kovach K, Tuerk PW, et al Randomized controlled trial of a nationally available weight control program tailored for adults with type 2 diabetes. Obesity 2016;24:2269–77. 10.1002/oby.21616 27804264

[24] Herdman M, Gudex C, Lloyd A, et al Development and preliminary testing of the new five-level version of EQ-5D (EQ-5D-5L). Qual Life Res 2011;20:1727–36. 10.1007/s11136-011-9903-x 21479777 PMC3220807

[25] Devlin NJ, Shah KK, Feng Y, et al Valuing health-related quality of life: an EQ-5D-5L value set for England. Health Econ 2018;27:7–22. 10.1002/hec.3564 28833869 PMC6680214

[26] Al-Janabi H, Flynn TN, Coast J Development of a self-report measure of capability wellbeing for adults: the ICECAP-A. Qual Life Res 2012;21:167–76. 10.1007/s11136-011-9927-2 21598064 PMC3254872

[27] Flynn TN, Huynh E, Peters TJ, et al Scoring the Icecap-a capability instrument. estimation of a UK general population tariff. Health Econ 2015;24:258–69. 10.1002/hec.3014 24254584 PMC4322472

[28] Doherty A, Jackson D, Hammerla N, et al Large scale population assessment of physical activity using wrist worn accelerometers: the UK Biobank study. PLoS One 2017;12:e0169649 10.1371/journal.pone.0169649 28146576 PMC5287488

[29] Wareham NJ, Jakes RW, Rennie KL, et al Validity and repeatability of the EPIC-Norfolk physical activity questionnaire. Int J Epidemiol 2002;31:168–74. 10.1093/ije/31.1.168 11914316

[30] Golubic R, May AM, Benjaminsen Borch K, et al Validity of electronically administered recent physical activity questionnaire (RPAQ) in ten European countries. PLoS One 2014;9:e92829 10.1371/journal.pone.0092829 24667343 PMC3965465

[31] Mulligan AA, Luben RN, Bhaniani A, et al A new tool for converting food frequency questionnaire data into nutrient and food group values: fetA research methods and availability. BMJ Open 2014;4:e004503 10.1136/bmjopen-2013-004503 PMC3975761 24674997

[32] O'Neill J, Tabish H, Welch V, et al Applying an equity lens to interventions: using progress ensures consideration of socially stratifying factors to illuminate inequities in health. J Clin Epidemiol 2014;67:56–64. 10.1016/j.jclinepi.2013.08.005 24189091

[33] Griffin SJ, Borch-Johnsen K, Davies MJ, et al Effect of early intensive multifactorial therapy on 5-year cardiovascular outcomes in individuals with type 2 diabetes detected by screening (ADDITION-Europe): a cluster-randomised trial. Lancet 2011;378:156–67. 10.1016/S0140-6736(11)60698-3 21705063 PMC3136726

[34] White IR, Thompson SG Adjusting for partially missing baseline measurements in randomized trials. Stat Med 2005;24:993–1007. 10.1002/sim.1981 15570623

[35] Bradley C, Todd C, Gorton T, et al The development of an individualized questionnaire measure of perceived impact of diabetes on quality of life: the ADDQoL. Qual Life Res 1999;8:79–91. 10.1023/A:1026485130100 10457741

[36] Dalton M, Finlayson G, Hill A, et al Preliminary validation and principal components analysis of the control of eating questionnaire (CoEQ) for the experience of food craving. Eur J Clin Nutr 2015;69:1313–7. 10.1038/ejcn.2015.57 25852028

[37] Westenhoefer J, Stunkard AJ, Pudel V Validation of the flexible and rigid control dimensions of dietary restraint. Int J Eat Disord 1999;26:53–64. 10.1002/(SICI)1098-108X(199907)26:1<53::AID-EAT7>3.0.CO;2-N 10349584

[38] Gormally J, Black S, Daston S, et al The assessment of binge eating severity among obese persons. Addict Behav 1982;7:47–55. 10.1016/0306-4603(82)90024-7 7080884

[39] Grupski AE, Hood MM, Hall BJ, et al Examining the binge eating scale in screening for binge eating disorder in bariatric surgery candidates. Obes Surg 2013;23:1–6. 10.1007/s11695-011-0537-4 23104387 PMC4874644

