# Supplementary material for: Clinical and cost-effectiveness of a diabetes education and behavioural weight management programme versus a diabetes education programme in adults with a recent diagnosis of type 2 diabetes: study protocol for the Glucose Lowering through Weight management (GLoW) randomised controlled trial
Source: BMJ Open. 2020 Apr 28;10(4):e035020. doi: 10.1136/bmjopen-2019-035020 (PMC7213851; doi:10.1136/bmjopen-2019-035020)
Supplement: Author's manuscript [file bmjopen-2019-035020.draft_revisions.pdf]

BMJ Open is committed to open peer review. As part of this commitment we make the peer review history of every article we publish publicly available.

When an article is published we post the peer reviewers' comments and the authors' responses online. We also post the versions of the paper that were used during peer review. These are the versions that the peer review comments apply to.

The versions of the paper that follow are the versions that were submitted during the peer review process. They are not the versions of record or the final published versions. They should not be cited or distributed as the published version of this manuscript.

BMJ Open is an open access journal and the full, final, typeset and author-corrected version of record of the manuscript is available on our site with no access controls, subscription charges or pay-per-view fees (http://bmjopen.bmj.com).

If you have any questions on BMJ Open's open peer review process please email info.bmjopen@bmj.com

BMJ Open

The clinical and cost-effectiveness of a diabetes education and behavioural weight management programme versus a diabetes education programme in adults with a recent diagnosis of type 2 diabetes: Study protocol for the Glucose Lowering through Weight Management (GLoW) randomised controlled trial.

Journal:	*BMJ Open*
Manuscript ID	bmjopen-2019-035020
Article Type:	Protocol
Date Submitted by the Author:	18-Oct-2019
Complete List of Authors:	Ahern, Amy L.; University of Cambridge School of Clinical Medicine, MRC Epidemiology Unit<br>Woolston, Jenny; University of Cambridge School of Clinical Medicine, MRC Epidemiology Unit<br>Wells, Emma; University of Cambridge School of Clinical Medicine, MRC Epidemiology Unit<br>Sharp, Stephen; University of Cambridge School of Clinical Medicine, MRC Epidemiology Unit<br>Islam, Nazrul; University of Cambridge School of Clinical Medicine, MRC Epidemiology Unit<br>Lawlor, Emma; University of Cambridge School of Clinical Medicine, MRC Epidemiology Unit<br>Duschinsky, Robbie; University of Cambridge Primary Care Unit<br>Hill, Andrew; University of Leeds, School of Medicine<br>Doble, Brett; Duke-NUS Medical School<br>Wilson, Ed; University of East Anglia, Norwich Medical School<br>Hughes, Carly; Fakenham Medical Practice; University of East Anglia Faculty of Medicine and Health Sciences<br>Brennan, Alan; The University of Sheffield, School of Health and Related Research<br>Bostock, Jennifer; Patient and Public Involvement Representative<br>Boothby, Clare; University of Cambridge School of Clinical Medicine, MRC Epidemiology Unit<br>Griffin, Simon; University of Cambridge School of Clinical Medicine, MRC Epidemiology Unit; University of Cambridge School of Clinical Medicine, Primary Care Unit, Institute of Public Health
Keywords:	Diabetes & endocrinology < INTERNAL MEDICINE, PRIMARY CARE, General diabetes < DIABETES & ENDOCRINOLOGY, GENERAL MEDICINE (see Internal Medicine)

SCHOLARONE™
Manuscripts

I, the Submitting Author has the right to grant and does grant on behalf of all authors of the Work (as defined in the below author licence), an exclusive licence and/or a non-exclusive licence for contributions from authors who are: i) UK Crown employees; ii) where BMJ has agreed a CC-BY licence shall apply, and/or iii) in accordance with the terms applicable for US Federal Government officers or employees acting as part of their official duties; on a worldwide, perpetual, irrevocable, royalty-free basis to BMJ Publishing Group Ltd ("BMJ") its licensees and where the relevant Journal is co-owned by BMJ to the co-owners of the Journal, to publish the Work in this journal and any other BMJ products and to exploit all rights, as set out in our licence.

The Submitting Author accepts and understands that any supply made under these terms is made by BMJ to the Submitting Author unless you are acting as an employee on behalf of your employer or a postgraduate student of an affiliated institution which is paying any applicable article publishing charge ("APC") for Open Access articles. Where the Submitting Author wishes to make the Work available on an Open Access basis (and intends to pay the relevant APC), the terms of reuse of such Open Access shall be governed by a Creative Commons licence – details of these licences and which Creative Commons licence will apply to this Work are set out in our licence referred to above.

Other than as permitted in any relevant BMJ Author's Self Archiving Policies, I confirm this Work has not been accepted for publication elsewhere, is not being considered for publication elsewhere and does not duplicate material already published. I confirm all authors consent to publication of this Work and authorise the granting of this licence.

For peer review only

V4 16.09..2019

The clinical and cost-effectiveness of a diabetes education and behavioural weight management programme versus a diabetes education programme in adults with a recent diagnosis of type 2 diabetes: Study protocol for the Glucose Lowering through Weight Management (GLoW) randomised controlled trial.

Dr Amy L. Ahern[1*], Mrs Jenny Woolston[1], Ms Emma Wells[1], Mr Stephen J. Sharp[1], Dr Nazrul Islam[1], Dr Emma Lawlor[1], Dr Robbie Duschinsky[2], Professor Andrew J. Hill[3], Dr Brett Doble[4], Dr Edward Wilson[5], Professor Stephen Morris[2], Dr Carly Hughes [5,6], Professor Alan Brennan[7], Mrs Jennifer Bostock, Dr Clare E. Boothby[1], Professor Simon J. Griffin[1,2]

1. MRC Epidemiology Unit, Box 285 Institute of Metabolic Sciences, School of Clinical Medicine, University of Cambridge, Cambridge, CB2 0QQ, UK
2. The Primary Care Unit, Institute of Public Health, School of Clinical Medicine, University of Cambridge, Forvie Site, Cambridge, CB2 OSR, UK
3. School of Medicine, University of Leeds, Worsley Building, Leeds, LS2 9JT, UK
4. Duke-NUS Medical School, College Road, Singapore 169857
5. Norwich Medical School, University of East Anglia, Norwich Research Park, Norwich, NR4 7TJ, UK
6. The Fakenham Medical Practice, Fakenham, UK
7. School of Health and Related Research, University of Sheffield, Regent Court, Regent Street, Sheffield, SD1 4DA, UK

* Corresponding author: MRC Epidemiology Unit, University of Cambridge, Box 285 Institute of Metabolic Science, Cambridge Biomedical Campus, Cambridge, UK, CB2 0QQ, ala34@cam.ac.uk

For peer review only

For peer review only - http://bmjopen.bmj.com/site/about/guidelines.xhtml

Abstract

Introduction: People with type 2 diabetes can improve glycaemic control or even achieve remission through weight loss, and reduce their use of medication and risk of cardiovascular disease. The Glucose Lowering through Weight management (GLoW) trial will evaluate whether a tailored diabetes education and behavioural weight management programme is more effective and cost-effective than a diabetes education programme in helping people with overweight or obesity and a recent diagnosis of type 2 diabetes to lower their blood glucose, lose weight, and improve other markers of cardiovascular risk.

Methods and Analysis: Pragmatic, randomised, single-blind, parallel group, two-arm, superiority trial. We will recruit 576 adults with BMI>25kg/m^2 and diagnosis of type 2 diabetes in the past 3 years, and randomise them to a tailored diabetes education and behavioural weight management programme or a diabetes education programme. Participants will attend measurement appointments at a local GP practice or research centre at baseline, 6 and 12 months. The primary outcome is 12 month change in glycated haemoglobin. The effect of the intervention on the primary outcome will be estimated and tested using a linear regression model (ANCOVA) including randomisation group and adjusted for baseline value of the outcome and the randomisation stratifiers. Participants will be included in the group to which they were randomised, under the intention-to-treat principle. Secondary outcomes include 6 and 12 month changes in body weight, body fat percentage, systolic and diastolic blood pressure, and lipid profile; probability of achieving good glycaemic control; probability of achieving remission from diabetes; probability of losing 5% and 10% body weight; and modelled cardiovascular risk (UKPDS). An intention-to-treat within-trial cost effectiveness analysis will be conducted from NHS and societal perspectives using participant-level data. Qualitative interviews will be conducted with participants to understand why and how the programme achieved its results and how participants manage their weight after the programme ends.

Ethics and Dissemination: Ethical approval was received from East of Scotland Research Ethics Service on 15th May 2018 (18/ES/0048). This protocol (version 3) was approved on 19th June 2019.

For peer review only - http://bmjopen.bmj.com/site/about/guidelines.xhtml

Findings will be published in peer-reviewed scientific journals and communicated to other stakeholders as appropriate.

Trial registration: ISRCTN18399564, registered 9th July 2018

Keywords

Weight management, type 2 diabetes, diabetes education, primary care, randomised controlled trial

Strengths and Limitations

- This trial will provide robust evidence of the clinical and cost-effectiveness of a scalable tailored diabetes education and behavioural weight management programme versus diabetes education alone in adults with a recent diagnosis of type 2 diabetes.
- If shown to be cost-effective, the intervention being evaluated already has the appropriate infrastructure to be readily integrated into existing UK care pathways and delivered to large numbers of people.
- The behavioural programme is already widely available across many countries and this model of care could be readily adopted across health care services internationally.
- The trial only includes follow up at 6 months and 12 months and longer term data may be needed to understand the longer term impact of these programmes. However, consent has been obtained to follow up participants through national registries and medical records for up to 15 years.

For peer review only

Background

The treatment of diabetes and related complications (e.g. cardiovascular disease, amputation, kidney failure) uses approximately10% of the UK NHS budget.(1) This is predicted to rise to 17% in 2035 as the number of people with diabetes in the UK rises to 6.25 million, of which 5.6 million cases will be adults with type 2 diabetes (T2D).(1) Adults who are living with T2D are at increased risk of developing physical and mental health comorbidities, and have reduced quality of life and shorter life expectancy.(2,3) There are considerable social and economic costs to the individual living with diabetes, as well as to wider society.(1,2,4)

While T2D is typically characterised as a progressive, irreversible condition, there is evidence of remission in patients losing weight through bariatric surgery(5,6) or closely supervised very-low-calorie formula diets.(7)·(8) However, many patients with T2D may be unsuitable for or unwilling to undergo these interventions and, given their high cost and reliance on specialists, they are unlikely to be widely adopted in the NHS in the near future. Partial or complete remission of type 2 diabetes has also been observed following smaller weight losses achieved through behavioural interventions.(9) Moreover, even without remission, weight loss and behaviour change can produce improvements in health outcomes for people who have diabetes. We have shown that losing a moderate amount of weight or making healthy behaviour changes (e.g. increasing physical activity, reducing alcohol, energy and fat intake) in the first year after diagnosis can reduce the likelihood of stroke or heart attack in the next 5-10 years(10–12) and increase the likelihood of achieving remission at 5 years.(13)

The Look AHEAD trial demonstrated that intensive specialist-led behavioural programmes could lead to weight loss and reductions in cardiovascular risk factors over 8 year follow up.(14) However, there are currently insufficient resources in the UK NHS to provide intensive, specialist-led behavioural programmes to the 3.2 million individuals who have T2D and the additional 200,000 who are diagnosed each year. Instead, current guidelines focus on structured diabetes education and dietary advice,(15) which is cheaper and scalable but has small, short-term effects on weight and glycaemia, and relatively poor uptake.(16–18) A recent systematic review found that supportive behaviour-change

For peer review only - http://bmjopen.bmj.com/site/about/guidelines.xhtml

programmes (with ≥11 hours of contact time) achieve greater reductions in weight and HbA_{1c} than structured education without additional support (≤10hrs).(17) Integrating effective but scalable behaviour change programmes into care pathways for T2D could potentially improve patients' glycaemic control and related risk factors and reduce complications. This would improve health and quality of life for people living with diabetes and reduce the burden of diabetes on health care resources.

We have previously shown that commercial open-group behavioural weight management programmes, such as WW (formerly Weight Watchers®) or Slimming World, offer a scalable and cost-effective way to help people lose weight and reduce risk of diabetes.(19–22) A randomised trial in the US showed that a combination of WW classes and remote dietary counselling achieved greater weight losses and reductions in HbA_{1c} than standard care over 1 year in people with diabetes.(23) A quarter of participants randomised to this programme achieved good glycaemic control (HbA_{1c} below 53mmol/mol) at 12 months, compared with 14% of those receiving standard care. In the UK, a similar intervention has been developed for use in the NHS that combines referral to WW with NICE-compliant diabetes education and dietary advice. However, this programme is unlikely to be widely commissioned without robust evidence of cost-effectiveness. The proposed trial will provide reliable evidence on the relative effectiveness and cost-effectiveness of a tailored diabetes education and behavioural weight management programme vs diabetes education, for people with a recent diagnosis of T2D (≤3years).

Objectives

Primary Objective

To evaluate the effect of a tailored diabetes education and behavioural weight management programme (DEW) vs diabetes education (DE) on glycated haemoglobin (HbA_{1c}) at 12 months in adults with a recent diagnosis of type 2 diabetes.

For peer review only - http://bmjopen.bmj.com/site/about/guidelines.xhtml

Secondary Objectives

To evaluate the effect of DEW vs DE on:

- body weight, body fat percentage, blood pressure, lipid profile, and modelled 10 year cardiovascular risk at 6 and 12 months
- probability of achieving clinically significant weight loss, good glycaemic control or diabetes remission at 6 and 12 months
- changes in diet and physical activity at 6 and 12 months
- psychosocial factors associated with successful weight control at 6 and 12 months.

To evaluate the within-trial cost-effectiveness of DEW vs DE.

To assess the extent to which the two programmes reach the target population.

To explore participant and practitioner experiences of the two programmes and the extent to which these programmes meet their needs.

To clarify causal mechanisms, and identify contextual factors associated with variations in outcome.

To identify barriers and facilitators to maintenance of weight management behaviours after treatment cessation.

Methods and Analysis

Trial Design

This is a pragmatic, randomised, single-blind, parallel group, two-arm, superiority trial. Participants will be randomised to either the tailored diabetes education and behavioural weight management programme or to diabetes education. Block randomisation will be used with a 1:1 allocation stratified by sex and duration of diabetes (Figure 1).

For peer review only - http://bmjopen.bmj.com/site/about/guidelines.xhtml

Study Setting

Research active primary care practices in the East of England that currently refer patients with type 2 diabetes (≤3 years duration) to local structured diabetes education (DESMOND) and have active WW groups in the local community.

Participants

Participants will be 576 adults with overweight or obesity who have a diagnosis of type 2 diabetes in the past 3 years.

Inclusion criteria

- BMI ≥25kg/m^2
- Age ≥18 years
- Diagnosis of type 2 diabetes within the previous 36 months (confirmatory blood test will not be required)
- Capable of giving informed consent
- Have a good understanding of the English language (study materials are not tailored to support non-English language speakers)
- Willing to be randomised
- Willing to attend follow up visits at a local participating GP practice or research centre

Exclusion criteria

- Using insulin
- Previous/planned bariatric surgery
- Current/planned pregnancy
- Current diagnosis of eating disorder
- Already received a structured diabetes education programme

For peer review only - http://bmjopen.bmj.com/site/about/guidelines.xhtml

- GP considers unsuitable
- Participation in another structured behaviour change programme for diet and/or physical activity within the past 3 months

Interventions

Tailored diabetes education and behavioural weight management programme

The tailored structured education and behavioural weight management programme was developed and is delivered by WW. It lasts 6 months and is overseen by a registered dietitian with experience in diabetes, diet and behaviour counselling and specific training in the standard WW programme.

Structured diabetes education

The structured diabetes education programme is delivered remotely (video conferencing /telephone) on a 1:1 basis by the registered dietitian over two education sessions, 3-5 weeks apart (total time 1 hr 30 minutes, divided between 2 calls). It covers a standardised QISMET-accredited core curriculum but can then be adapted for the individual's needs. Additional self-help education materials to support the curriculum will be available online and are delivered to all participants via email or mail, at the patient's preference. These materials may be minimally tailored to the individual participant. Information on the materials can be found at www.weightwatchers.com/uk/live-well-diabetes. During the education sessions, materials on goal setting, understanding diabetes, the glycaemic index, carbohydrates, physical activity, and weight management are reviewed.

Behavioural programme

The behavioural weight management component consists of free membership of WW for 6 months. This includes attendance at weekly in-person group meetings (30-40 minutes) held at a variety of times in a range of local community venues. These are open-group meetings (new people may join or leave the group at any time) and are led by a coach (trained lay person with experience of the programme). Meetings include a confidential weigh in with the coach and a 30-minute interactive

For peer review only - http://bmjopen.bmj.com/site/about/guidelines.xhtml

205 education session led by the coach that includes advice on diet, physical activity, and positive mindset, using behavioural strategies (e.g. goal setting, self-monitoring, problem solving, modifying the personal food environment, and relapse prevention). Peer support is available from coaches and other group members. Participants can be accompanied by a friend, relative or carer. Participants will also have access to the WW app, online digital tools, and standard materials such as recipe booklets, physical activity guidance and meal trackers for the duration of the intervention. Participants can contact their coach for support/advice between meetings via an online chat function.

Participant engagement

Once a referral is received, the registered dietitian will telephone the participant, welcome them to the programme, sign them up to a local WW meeting, and arrange the first education session. Participants are also given an information sheet with details of how to contact the dietitian directly. A closed social media support group will be formed and all participants will be encouraged to join if they wish to do so. Activity on the group forum will be monitored and supported by the registered dietitian. Each week, the dietitian will contact 2-5 participants (selected at random from those who have participated in the programme for longer than 3 months) via the social media group or phone. Participants can also contact the dietitian proactively during the intervention period for additional remote support where needed, but will be encouraged to do so via the social media group in the first instance. If participants miss 4 or more WW standard in-person meetings their local coach will call to help the individual return to the programme.

Diabetes Education

In the East of England, the diabetes education programme most commonly commissioned by the Clinical Commissioning Group (CCG) is the Diabetes and Education Self-Monitoring for Ongoing and Newly Diagnosed (DESMOND) programme.(16,18) This is a structured diabetes education programme for people with a recent diagnosis of type 2 diabetes (≤3 years since diagnosis).

For peer review only - http://bmjopen.bmj.com/site/about/guidelines.xhtml

Participants can attend 6 hours of structured self-management group education, covering: thoughts and feelings about diabetes; understanding diabetes and glucose - what happens in the body; understanding risk factors and complications associated with diabetes; understanding monitoring and medication; how to take control - food choices and physical activity; and planning for the future. The structured education is delivered in 1 day or 2 half days by two trained health care professionals in local health care or community venues. Sessions are delivered in groups of up to 10 participants, and participants can bring a friend or partner with them. The education sessions are supported by specially developed resources.

Outcomes

Primary Outcome

12 month change from baseline in glycated haemoglobin (HbA_{1c}).

Secondary Outcomes

6 month change from baseline in HbA_{1c}.

6 and 12 month changes from baseline in body weight, body fat percentage, systolic and diastolic blood pressure, total cholesterol, HDL cholesterol, and LDL cholesterol.

Good glycaemic control (HbA_{1c} <53mmol/mol) at 6 and 12 months.

Remission from diabetes (HbA_{1c} <48mmol/mol and without glucose-lowering medication for ≥2 months) at 6 and 12 months.

Weight loss ≥5% and ≥10% of initial body weight at 6 and 12 months.

Modelled cardiovascular risk (UKPDS) at 12 months.

For peer review only - http://bmjopen.bmj.com/site/about/guidelines.xhtml

Behavioural and Psychosocial Outcomes

6 and 12 month changes from baseline in objectively measured physical activity (accelerometry), self-reported physical activity, objective marker of fruit and vegetable intake (plasma carotenoids), and self-reported dietary intake.

6 and 12 months changes from baseline, adjusted for baseline, in dietary restraint, control over food cravings, emotional eating, self-regulatory skills, social support and diabetes related quality of life.

Health Economic Outcomes

Detailed micro-costing of DEW and DE.

Health and social care resource use over 12 months (medical notes, registry data, resource use questionnaire).

Self-reported out-of-pocket costs and lost productivity (e.g. due to days off work, up to 12 months).

12 month quality-adjusted life-years (QALYs) based on Health Related Quality of Life (HRQoL) (EQ-5D-5L)(24,25) and capability/wellbeing (ICECAP-A)(26,27)

Total and incremental costs from NHS and societal perspectives; incremental net (monetary) benefit; incremental cost-effectiveness and cost-utility ratios; value of information estimates.

Uptake and adherence

Number and characteristics of participants who:

- are offered the opportunity to participate in the trial
- enrol in the trial
- attend the intervention
- adhere to the intervention (including specific intervention components)

For peer review only - http://bmjopen.bmj.com/site/about/guidelines.xhtml

A detailed protocol for process evaluation will also be developed.

Visits and Measurements

Visit Schedule

Participants will be asked to attend measurement appointments at a participating primary care practice or research centre at 0, 6, and 12 months. Details of measures at each assessment are summarised in Figure 2. Participants will be reimbursed for reasonable travel expenses and given an honorarium for attending measurement appointments (£10 for baseline and 6 month visits, £30 for the 12 month visit). Honoraria for assessment attendance are not dependent on intervention attendance/completion.

Anthropometric Measures

Anthropometric measurements will be made at participating primary care practices or research centres by research-trained health care professionals (hereafter "research nurses") blind to intervention allocation, in line with standardised operating procedures. Participants will be asked to remove shoes and heavy clothing items. Height will be measured in cm using a mounted stadiometer (make and model dependent on practice). Where possible, weight (kg) and body fat percentage will be measured using a calibrated Tanita segmental body composition analyser (Tanita Ltd; MA Tokyo, Japan; model dependent on practice), which will be provided to practices by the research team. Where we have an insufficient number of Tanita scales to measure body fat at a practice, we will measure weight (kg) only, using calibrated electronic scales (model dependent on practice). To maximise the number of participants for whom we can measure body fat, we will prioritise the use of Tanitas at large practices with a large number of eligible patients, and CRN-led practices (as CRN nurses can transport the Tanita scales to numerous practices). Blood pressure will be measured 3 times in a resting state using a calibrated OMRON automatic blood pressure monitor (OMRON Healthcare UK Ltd: Milton Keynes, UK; model dependent on practice).

For peer review only

For peer review only - http://bmjopen.bmj.com/site/about/guidelines.xhtml

If a research nurse is not available to conduct follow up assessments, assessments may be conducted by trained research centre staff. If participants are unable to attend follow up appointments at a participating practice, the research team may offer appointments at the local hospital or research centre, or home visits. Participants who are unable or unwilling to attend a visit will be asked to provide a self-measured weight. All other participants will also be asked for a self-measured weight at the time of appointment booking, to enable us to quantify the degree of misreporting of self-measured weights.

Biochemical Measures

Participants will be asked to provide a blood sample for the measurement of HbA_{1c}, lipid profile, and carotenoids. If possible, this will be taken at the practice at the same time as the anthropometric measurements. Blood samples will be collected in three tubes labelled with an anonymised barcode (4.9ml serum tube for lipid profile, 2.6ml EDTA tube for $HbA_{1c,}$ 4.9ml lithium heparin (LH) tube for carotenoids). The LH tube is light sensitive, so will be wrapped in foil to avoid degradation. Samples will be placed in a Royal Mail Safebox™, and posted via first class mail to the central laboratory for analysis. Each sample is stable for up to 3 days at room temperature. Plasma samples for analysis of carotenoids will be frozen and analysed in batches.

Behavioural Measures

Physical Activity

Physical activity will be measured objectively using a triaxial accelerometer (Axivity AX3, Newcastle, UK) which measures raw acceleration (m/s^2). Participants will be asked to wear the accelerometer on their non-dominant wrist continuously for 7 consecutive days and nights. The device is small, light and waterproof and is worn like a watch. It does not need to be taken off if showering, bathing or swimming.

For peer review only

For peer review only - http://bmjopen.bmj.com/site/about/guidelines.xhtml

At the baseline visit, the nurse will take the participant through the accelerometer instruction sheet. They will demonstrate to the participant how they should wear the monitor on their wrist. The nurse will also ensure that the timing of the monitor and return is feasible for the participant. The study team will initialise the accelerometer and will post it out to the participant with the instruction sheet. The participant will wear the monitor for 7 days and nights and will complete an activity log. For the 6 and 12 month visits, accelerometers will be mailed out to participants. At the end of 7 days the participant will be asked to post the device back to the research centre using pre-paid, pre-addressed materials. Trained staff will download the data from the accelerometer and check it to ensure the integrity of the data recording. This method has been used in large-scale epidemiological studies including the UK Biobank study in 100,000 individuals.(28) Data will be analysed using established methods used in UK Biobank. The outcome variables of interest are vector magnitude, a measure of total physical activity, and intensity distribution, from which duration of time in different levels of physical activity can be inferred.

Self-reported physical activity will be measured using the Recent Physical Activity Questionnaire (RPAQ), a validated measure of four domains of physical activity (leisure time, occupation, commuting, and domestic life) that has been used in a number of intervention studies and large-scale epidemiological studies. (29,30) This will be administered as part of the self-report questionnaires.

Diet

Plasma carotenoids will be measured as an objective marker of fruit and vegetable intake. Self-reported dietary intake will be measured at each timepoint using a version of the previously validated EPIC Food Frequency Questionnaire (FFQ)(31) that has been adapted for a 6 month recall period. This will be administered as part of the self-report questionnaires

Self-report Questionnaires

Participants will complete a demographics questionnaire at baseline based on Progress-Plus(32) factors (place of residency, race/ethnicity, occupation, gender/sex, religion, education, socioeconomic

For peer review only

For peer review only - http://bmjopen.bmj.com/site/about/guidelines.xhtml

status, social capital, age, disability, relationship status, caring responsibilities, car ownership, access to the internet).

Self-reported behavioural and psychosocial measures will be collected via validated self-report questionnaires, which can be completed on paper or online, at the participant's preference. A full list of questionnaires can be found in Table 1.

Intervention Adherence

Adherence data for the tailored diabetes education and behavioural weight management programme (including attendance at weekly WW meetings and remote education sessions, and use of online tools) will be collected by WW as part of an established system for NHS contracts. Self-reported intervention adherence for both programmes will also be collected via a self-report questionnaire.

Table 1: Questionnaires administered in the GLoW trial

**Domain**	**Measure**	**Time point**
Demographics	Bespoke questionnaire based on PROGRESS-Plus(32)	Baseline
Health-related quality of life	EQ-5D-5L(24,25)	All
Capability/Wellbeing	ICECAP-A(26,27)	All
Diabetes-related quality of life	Audit of Diabetes Dependent Quality of Life - ADDQoL(33)	All
Health/ Social Care Use	Bespoke resource use questionnaire	All
Food Cravings	Control of Eating Questionnaire(34)	All
Dietary Restraint	Three Factor Eating Questionnaire – Restraint subscale(35)	All
Binge Eating	The Binge Eating Scale (BES)(36,37)	All
Dietary Intake	Food Frequency Questionnaire(31)	All

For peer review only

For peer review only - http://bmjopen.bmj.com/site/about/guidelines.xhtml

Physical Activity	Recent Physical Activity Questionnaire (RPAQ)(29,30)	All
Intervention adherence	Bespoke questionnaire	6 months

364

365

366 ***Medical Notes Review***

367 Healthcare resource use will be obtained for all participants via medical notes review and registry
368 data. Primary care records will be used to extract numbers of visits to the GP and community health
369 care workers (defined as any primary or community based health worker such as nurse and allied
370 health professional contacts noted in the patient's record) and prescribed medications. Last recorded
371 weight, HbA_{1c} value, smoking status and diabetes status will also be extracted. This will help to
372 minimise missing data from missed assessment appointments.

373 The notes review will be carried out at each study visit and following the end of the study by a
374 research nurse blind to intervention allocation.

375 - Baseline visit: notes review to cover the 3 months prior to study start
376 - 6 month visit: notes review to cover from baseline up to 6 months
377 - 12 month visit: notes review to cover from 6 months to 12 months

378 We will obtain consent to conduct future notes reviews after the study has completed (up to 15 years
379 post study) for future studies of long term outcomes.

380

381 ***Administrative and Registry Data***

382 We will obtain consent at baseline for future collection of administrative and registry data on health
383 event and health care usage information (up to 15 years post-study). Outcomes will include hospital
384 episode statistics, cardiovascular events, stroke, myocardial infarction, cancer and death, and will be
385 obtained from NHS digital, the Healthcare Quality Improvement Partnership (HQIP) and the National
386 Cancer Registration and Analysis Service (NCRAS). This will allow us to evaluate the longer term
387 impact of these programmes on diabetes- and weight-related morbidity and mortality.

For peer review only - http://bmjopen.bmj.com/site/about/guidelines.xhtml

For peer review only

Qualitative Interviews

Around the time of the 6 month follow up, we will conduct semi-structured interviews with participants from both intervention arms (N ≥ 26). Participants will be recruited in a 2:1 ratio (DEW:DE), as we are interested in comparing the experience of the two groups, but we expect the group receiving the tailored programme to provide the richest data on the experiences of weight loss and weight loss maintenance. Participants will be purposively sampled (demographic characteristics, programme attendance/adherence, weight change). We will also conduct interviews with treatment providers (N ≥ 12) to understand their experience of delivering the intervention. Interviews will be conducted by a trained research associate and audio recorded.

Questions will focus on exploring:

- How did participants and practitioners experience the programme and its components: i) education; ii) WW meetings; iii) digital tools; iv) remote dietetic counselling?
- What were the causal mechanisms and contextual factors that were associated with different outcomes?
- What were the needs of participants at the end of the programme?
- What were the facilitators and barriers to maintaining behavioural change?

Recruitment and Enrolment

Recruitment of Practices

Primary care practices will be identified and recruited by Clinical Research Networks (CRN). Participating practices must be currently referring patients with a recent diagnosis of type 2 diabetes to structured diabetes education (DESMOND) as part of standard practice and should have active WW meetings in the local area. They should also be research active with the capacity to recruit and follow up participants. The CRN will attempt to recruit practices from diverse areas using known characteristics of the practice population, to enable recruitment of a sample broadly representative of

For peer review only - http://bmjopen.bmj.com/site/about/guidelines.xhtml

the target population (UK adults with overweight and obesity who have a recent diagnosis of type 2 diabetes).

Patient identification

Eligible patients will be identified through electronic searches of primary care records and waiting lists for diabetes education. GPs will write to all potentially eligible patients inviting them to participate. This letter will include a participant information sheet and details of how to contact the study team at the research centre. Participants will also receive opportunistic invitations during routine consultations, via an invitation letter and participant information sheet to take home with them. Pop up alerts on patient records (triggered by diagnosis or attempted referral to diabetes education) will be implemented to facilitate opportunistic recruitment. To boost enrolment, advertisements will also be placed in local pharmacies, news media and other relevant settings.

Enrolment

Patients willing to participate will be asked to contact the research centre for more information. This can be by telephone, email, or reply slip. Research centre staff will answer any questions and conduct a telephone screening. If patients are eligible and willing to participate, the research staff will check that they have received the participant information sheet (sending another if they have not) and arrange a baseline assessment with the research nurse at a local participating practice or research centre.

At the baseline visit, the research nurse will take informed consent. This will include ensuring that participants have read the information sheet and had the opportunity to ask questions. They will also confirm eligibility, including an objective measure of height and weight, before taking the rest of the baseline measurements. At the end of each day, the research nurse will email the study coordinator a list of participants who have formally enrolled on the trial.

For peer review only

For peer review only - http://bmjopen.bmj.com/site/about/guidelines.xhtml

Randomisation

Randomisation sequence

Participants will be allocated to one of the two intervention arms in a 1:1 allocation using individual-level blocked randomisation stratified by sex (male, female) and duration of diabetes (<1 year, 1-3 years) with a block size of 6. The randomisation sequence will be computer-generated by the trial statistician and the randomisation process implemented by the data manager. The sequence will be unknown to all other personnel, including study coordinators, outcome assessors and investigators.

Method of implementing the allocation sequence

When participant eligibility has been confirmed at the baseline visit, the practice will inform the study coordinator. The study coordinator will enter the participant's details into the trial database, which will automatically assign an intervention to the participant.

The study coordinator (or member of their team) will write to the participant to inform them of their intervention allocation, and will also inform the participant's GP. The study coordinator or the GP (depending on practice and intervention allocation) will also send a referral form to the intervention provider, giving the details of the participant who has been referred to them.

Blinding

Given the nature of the intervention, it is not possible to blind participants to their intervention group. GPs will be informed of the intervention allocation and participants will be allowed to discuss the intervention with their GP, as they would outside a trial scenario. However, participants will be asked not to reveal their intervention group to outcome assessors (i.e. research nurses taking measurements). The trial statistician and the Investigators will be blinded to intervention allocation until the database is locked and the primary analysis complete.

For peer review only

For peer review only - http://bmjopen.bmj.com/site/about/guidelines.xhtml

Statistics and Data Analysis

Sample size calculation

The primary outcome is 12 month change from baseline in HbA_{1c}. Based on data from a previous trial in adults with a recent diagnosis of T2D,(38) we assumed a 16mmol/mol SD, a 0.8 correlation between baseline and follow-up and 25% attrition. In a US trial of a similar intervention in people with T2D of any duration, a difference of 4mmol/mol was observed between intervention (-3mmol/mol) and control (+1mml/mol) at 12 months.(23) We need 576 participants to detect a difference between randomised groups of 3mmol/mol HbA_{1c} with 90% power at a 5% significance level.

Statistical analysis plan

A detailed statistical analysis plan will be developed and signed off by the Trial Steering Committee prior to analysis. Participants will be analysed in the group to which they were randomised, based on the intention-to-treat principle. The intervention effect, representing the baseline-adjusted difference in change from baseline to 12 months in HbA_{1c} between the intervention and control group, will be estimated using a linear regression model including randomisation group, baseline value of HbA_{1c} (i.e. analysis of covariance, ANCOVA), and the randomisation stratifiers (sex, duration of diabetes). The missing indicator method(39) will be used to ensure inclusion of participants with a missing baseline value of HbA_{1c}. Participants with missing values of HbA_{1c} at 12 months will be excluded (i.e. a complete-case analysis). If there are >5% of participants with missing values of HbA_{1c} at 12 months, a sensitivity analysis will be performed using multiple imputation by chained equations; full details of this analysis will be provided in the statistical analysis plan.

Continuous secondary outcomes will be analysed using the method described above. Binary secondary outcomes will be analysed using a logistic regression model including randomisation group and the randomisation stratifiers.

For peer review only - http://bmjopen.bmj.com/site/about/guidelines.xhtml

For the primary outcome, effect modification by (1) sex, (2) index of multiple deprivation (high, low), (3) educational qualification (below post-secondary, post-secondary and above), (4) duration of diabetes (<1 year; 1-3 years) will be tested using an F-test of the relevant multiplicative interaction parameter in the ANCOVA model. If the p-value for a particular interaction is <0.05, then the intervention effect and 95% CI will be estimated within the relevant subgroups.

Economic evaluation

A detailed health economics analysis plan will be developed and signed off by the Trial Steering Committee prior to analysis. An intention-to-treat within-trial cost effectiveness analysis will be conducted from both the NHS and the societal perspectives using participant-level data. Benefits will be measured by changes in HbA_{1c} at 12 months for the primary analysis and changes in weight (kg), HRQoL (EQ5D-5L), capability/wellbeing (ICECAP-A), and quality-adjusted life years at 12 months for the secondary analyses. Resource use data will be extracted from patient-completed questionnaires and validated with primary care notes review. Unit costs will be extracted from standard NHS cost databases and publications (e.g. NHS Reference Costs). Costs and benefits will be left undiscounted as follow up is only one-year. If required and appropriate, missing data will be imputed using recognised techniques such as multiple imputation. Descriptive statistics for resource use, total costs, HRQoL, and capability/wellbeing at 1 year will be reported as well as incremental cost-effectiveness and cost-utility ratios and incremental net (monetary) benefit measures. We will undertake deterministic and probabilistic sensitivity analyses, presenting the results of the latter as cost-effectiveness acceptability curves. Value of information analysis will quantify the value of reducing the decision uncertainty which will inform whether further research is worthwhile following completion of this study, and if so on which parameters.

Qualitative Evaluation

For peer review only - http://bmjopen.bmj.com/site/about/guidelines.xhtml

Qualitative analyses will explore how the programmes were implemented; participant and provider perceptions of the extent to which the programmes met patient needs; and factors participants and providers regarded as causally significant. Analyses will also explore the key challenges anticipated around treatment cessation and weight loss maintenance, and the value attached to the possibility of freedom from or reduction in medication and the concept of "remission".

Recordings will be transcribed by an experienced external agency and checked for accuracy by the research team. Verbatim transcripts will be coded using NVivo software, retaining a focus on narrative sequences and transitions as well as salient themes. A dual coding approach will be used: a first inductive round based on emerging themes relating to the research questions; a second round sensitised by quantitative findings. In the first inductive stage, open codes will be generated based on line-by-line scrutiny of verbatim transcripts uploaded into NVivo. Inconsistencies between coders will be resolved through discussion. Patient and Public Involvement (PPI) representatives will assist with the analysis of qualitative data; this will include coding a sub-sample of transcripts following training, and ensuing dialogue.

Trial Steering Committee

The Trial Steering Committee (TSC) will provide overall supervision for the GLoW Trial on behalf of the Trial Sponsors (NHS Cambridgeshire and Peterborough CCG, University of Cambridge) and Trial Funder (NIHR Clinical Commissioning Facility), and ensure that the project is conducted to the rigorous standards set out in the UK Policy Framework for Health and Social Care Research and the Guidelines for Good Clinical Practice. The TSC will provide advice to the Investigators on all aspects of the trial and will review and agree the trial protocol, the statistical analysis plan, and any amendments to the protocol. The TSC will be chaired by Professor Andrew Farmer (University of Oxford). Independent members include Professor Lucy Yardley (University of Southampton), Dr Thomas Fanshawe (University of Oxford), Dr Edel Doherty (NUI Galway), Mr Graham Rhodes (PPI

For peer review only - http://bmjopen.bmj.com/site/about/guidelines.xhtml

representative) and Ms Hazel Patel (PPI representative). This is a low risk trial in which participants in both trial groups are referred to accredited education programmes. There are no rules for early stopping and participants and GPs are not blind to intervention allocation. Thus a separate data monitoring committee is not deemed to be necessary.

Patient and Public Involvement

Patient and Public Involvement (PPI) informs the design, management, analysis and dissemination of the GLoW study. The initial ideas and research proposal were reviewed by 3 members of Fakenham Weight Management Service and 6 members of the University of Cambridge PPI Panel. A PPI representative (Jennifer Bostock) is a member of our Investigator team and has contributed to the design of the protocol. She will also contribute to designing and delivering PPI training, preparing ethics and R&D submissions, co-authoring journal articles and the final report, disseminating findings to a wide range of audiences, and supporting other PPI members. Two PPI representatives are members of the Trial Steering Committee. They will review the final study reports and contribute to the writing of specific sections, such as the lay summary.

To maximise participant engagement and retention, and minimise burden, PPI representatives review the content, design, and delivery of participant-facing materials. PPI representatives will also support the design of the qualitative interview schedule and the analysis and interpretation of qualitative data. Including PPI perspectives in plans for dissemination will ensure that we access an appropriate range of audiences and communicate messages effectively. PPI representatives will advise on content and methods of dissemination and will review public facing documents such as newsletters and press releases.

PPI representatives will be reimbursed for their time and expenses in a timely manner, and tailored PPI training will be provided to suit the specific needs of the individual and their role.

For peer review only

For peer review only - http://bmjopen.bmj.com/site/about/guidelines.xhtml

Ethics and Dissemination

The NHS Cambridgeshire and Peterborough CCG and University of Cambridge are the co-sponsors of the trial. The protocol and all participant facing materials were reviewed by the East of Scotland Research Ethics Service. Initial approval was received on 15th May 2018 (18/ES/0048) and HRA approval was granted on 31st May 2018. This protocol (version 3) was approved on 19th June 2019. If needed, the Investigators will submit any proposed substantial and non-substantial amendments to the protocol or other approved documents to the HRA for approval. Substantial amendments will not be implemented until relevant approvals have been given. The MRC Epidemiology Unit has an over-arching data management policy (DMP) that encompasses the standards and processes applied to all research and operational activities in the Unit. A study-specific data management plan, based on this DMP, details how data will be collected, stored, transferred, accessed and archived. The PIs will ensure that all data generated, stored and shared from this trial will be handled in compliance with the DMP and the General Data Protection Regulations.

All specified analyses will be written up as scientific papers and submitted for publication in peer-reviewed open-access journals. Members of the research team will be involved in reviewing drafts of the manuscripts, abstracts, and any other publications arising from the trial. The Principal Investigators will have final approval on all publications and any press release, where appropriate. Authorship will be determined using ICMJE criteria. Upon publication of the main findings, participants will be sent a newsletter that describes the results and gives details of who to contact to ask questions or obtain further information. Newsletters will be prepared with input from PPI representatives. Where appropriate, we will communicate our findings to local and national stakeholders via tailored summaries of the key findings, and by presentations at meetings of local and national networks. Representatives from these groups will be involved in our research throughout and will support us in identifying opportunities for dissemination.

Trial Status

For peer review only - http://bmjopen.bmj.com/site/about/guidelines.xhtml

This protocol (version 3) was approved on 19th June 2019. Recruitment for the trial began on 6th August 2018 and is expected to close in April 2019.

Author Contributions

ALA and SJG are joint principal investigators. ALA, SJG, SJS, RD, SM, AH, BD, CH, AB and JB are grant holders. JW and EW are the trial coordinators. All authors contributed to the design of the protocol. ALA and JW wrote the first draft of the manuscript. All authors contributed to the writing and critical revision of the manuscript.

Funding and Support in Kind

This study is funded by the National Institute for Health Research (NIHR) Programme Grants for Applied Research Programme (Reference Number RP-PG-0216-20010). The views expressed are those of the author(s) and not necessarily those of the NIHR or the Department of Health and Social Care. The tailored diabetes education and behavioural weight management programme (Live Well With Diabetes) is provided by WW (formerly Weight Watchers) free of charge for the purposes of this trial. ALA, SJS and SJG are supported by the Medical Research Council (grant MC_UU_12015/4). The University of Cambridge has received salary support in respect of SJG from the NHS in the East of England through the Clinical Academic Reserve. This is an investigator-led trial. None of the funders have any role in the study design, the analysis and interpretation of data, or the writing of the report and decision to submit the report for publication.

Competing Interests Statement

ALA is principal investigator on another publically funded trial where WW provided the intervention at no cost but has received no personal fees. SJG reports grants from Medical Research Council, personal fees from Eli Lilly, and personal fees from Janssen, outside this programme of research. AJH reports personal fees from Slimming World and the College of Contemporary Health, outside this

For peer review only

For peer review only - http://bmjopen.bmj.com/site/about/guidelines.xhtml

programme of research. CH reports informal unpaid advice to Thriva. The other Investigators report no conflicts of interest.

Acknowledgements

We thank all staff from the MRC Epidemiology Unit Functional Group Team for input into the protocol design, particularly with regard to study coordination, anthropometry measurement, physical activity measurement, laboratory sample collection and analysis, data management, IT, business operations and research governance. We also thank NIHR CRN Eastern Primary Care research delivery colleagues, regional primary care stakeholders, and Cambridgeshire and Peterborough CCG Research Team for their input into the operational aspects of the study design and their comments on early iterations of the protocol.

Availability of data and materials

Following completion of the study and reporting of the main results, meta data will be made publically available via the lead institution's data sharing portal http://epi-meta.medschl.cam.ac.uk along with details of how to request data.

References

1. Hex N, Bartlett C, Wright D, Taylor M, Varley D. Estimating the current and future costs of Type1 and Type2 diabetes in the UK, including direct health costs and indirect societal and productivity costs. Diabetic Medicine. 2012;29(7):855–62.
2. HQIP, NHS, DUK. National Diabetes Audit , 2015-16 Report 2a : Complications and Mortality. 2017;(March).
3. Ali S, Stone MA, Peters JL, Davies MJ, Khunti K. The prevalence of co-morbid depression in adults with Type 2 diabetes: A systematic review and meta-analysis. Vol. 23, Diabetic Medicine. 2006. p. 1165–73.

For peer review only - http://bmjopen.bmj.com/site/about/guidelines.xhtml

4. Kanavos P, Aardweg S Van Den, Schurer W. Diabetes Expenditure, Burden of Disease and Management in 5 EU Countries. LSE Health [Internet]. 2012;(January):1–113. Available from: http://eprints.lse.ac.uk/54896/1/__libfile_REPOSITORY_Content_LSE Health and Social Care_Jan 2012_LSEDiabetesReport26Jan2012.pdf

5. Ribaric G, Buchwald JN, McGlennon TW. Diabetes and weight in comparative studies of bariatric surgery vs conventional medical therapy: A systematic review and meta-analysis. Obesity Surgery. 2014;24(3):437–55.

6. Sjöström L, Peltonen M, Jacobson P, Ahlin S, Andersson-Assarsson J, Anveden Å, et al. Association of bariatric surgery with long-term remission of type 2 diabetes and with microvascular and macrovascular complications. JAMA : the journal of the American Medical Association [Internet]. 2014;311(22):2297–304. Available from: http://www.ncbi.nlm.nih.gov/pubmed/24915261

7. Steven S, Hollingsworth K, Al-Mrabeh A, Avery L, Aribisala B, Caslake M, et al. Very-Low-Calorie Diet and 6 Months of Weight Stability in Type 2 Diabetes: Pathophysiologic Changes in Responders and Nonresponders. Diabetes care. 2016;March 21.

8. Lean MEJ, Leslie WS, Barnes AC, Brosnahan N, Thom G, McCombie L, et al. Primary care-led weight management for remission of type 2 diabetes (DiRECT): An open-label, cluster-randomised trial. The Lancet. 2017;

9. Gregg EW, Chen H, Wagenknecht LE, Clark JM, Delahanty LM, Bantle J, et al. Association of an intensive lifestyle intervention with remission of type 2 diabetes. JAMA : the journal of the American Medical Association [Internet]. 2012;308(23):2489–96. Available from: http://www.ncbi.nlm.nih.gov/pubmed/23288372

10. Long H G, Cooper AJM, Wareham J N, Griffin J S, Simmons K R. Healthy Behavior Change and Cardiovascular Outcomes in Newly Diagnosed Type 2 Diabetic Patients: A Cohort Analysis of the ADDITION-Cambridge Study. Diabetes Care [Internet]. 2014;37(6):1712–20. Available from: http://search.ebscohost.com/login.aspx?direct=true&db=jlh&AN=2012593245&site=ehost-

For peer review only

For peer review only - http://bmjopen.bmj.com/site/about/guidelines.xhtml

live

11. Strelitz J, Ahern AL, Long GH, Hare MJL, Irving G, Boothby CE, et al. Moderate weight change following diabetes diagnosis and 10 year incidence of cardiovascular disease and mortality. Diabetologia. 2019;

12. Strelitz J, Ahern AL, Long GH, Boothby CE, Wareham NJ, Griffin SJ. Changes in behaviors after diagnosis of type 2 diabetes and 10-year incidence of cardiovascular disease and mortality. Cardiovascular Diabetology. 2019;

13. Dambha-Miller H, Day AJ, Strelitz J, Irving G, Griffin SJ. Behaviour change, weight loss and remission of Type 2 diabetes: a community-based prospective cohort study. Diabetic Medicine. 2019;

14. Wadden TA. Eight-year weight losses with an intensive lifestyle intervention: The look AHEAD study. Obesity. 2014;22:5–13.

15. NICE NG28. Type 2 diabetes in adults: management. National Institute for Health and Care Excellence [Internet]. 2015;(December 2015):1–86. Available from: http://www.nice.org.uk/guidance/ng28/resources/type-2-diabetes-in-adults-management-1837338615493

16. Davies MJ, Heller S, Skinner TC, Campbell MJ, Carey ME, Cradock S, et al. Effectiveness of the diabetes education and self management for ongoing and newly diagnosed (DESMOND) programme for people with newly diagnosed type 2 diabetes: cluster randomised controlled trial. BMJ (Clinical research ed) [Internet]. 2008;336(7642):491–5. Available from: http://www.pubmedcentral.nih.gov/articlerender.fcgi?artid=2258400&tool=pmcentrez&rendertype=abstract

17. Pillay J, Armstrong MJ, Butalia S, Donovan LE, Sigal RJ, Vandermeer B, et al. Behavioral Programs for Type 2 Diabetes Mellitus: A Systematic Review and Network Meta-analysis. Annals of internal medicine [Internet]. 2015 Dec 1;163(11):848–60. Available from: http://annals.org/article.aspx?articleid=2446188%5Cnhttp://www.ncbi.nlm.nih.gov/pubmed/2641

For peer review only

For peer review only - http://bmjopen.bmj.com/site/about/guidelines.xhtml

4227

18. Gillett M, Dallosso HM, Dixon S, Brennan A, Carey ME, Campbell MJ, et al. Delivering the diabetes education and self management for ongoing and newly diagnosed (DESMOND) programme for people with newly diagnosed type 2 diabetes: cost effectiveness analysis. BMJ (Clinical research ed) [Internet]. 2010;341:c4093. Available from: http://www.pubmedcentral.nih.gov/articlerender.fcgi?artid=2924963&tool=pmcentrez&rendertype=abstract

19. Ahern AL, Wheeler GM, Aveyard P, Boyland EJ, Halford JCG, Mander AP, et al. Extended and standard duration weight-loss programme referrals for adults in primary care (WRAP): a randomised controlled trial. The Lancet. 2017;389(10085):2214–25.

20. Meads DM, Hulme CT, Hall P, Hill AJ. The cost-effectiveness of primary care referral to a UK commercial weight loss programme. Clinical obesity. 2014;4(6):324–32.

21. Fuller NR, Colagiuri S, Schofield D, Olson AD, Shrestha R, Holzapfel C, et al. A within-trial cost-effectiveness analysis of primary care referral to a commercial provider for weight loss treatment, relative to standard care--an international randomised controlled trial. International Journal of Obesity. England; 2013 Jun;37(6):828–34.

22. Jebb SA, Ahern AL, Olson AD, Aston LM, Holzapfel C, Stoll J, et al. Primary care referral to a commercial provider for weight loss treatment versus standard care: a randomised controlled trial. The Lancet [Internet]. 2011;378(9801):1485–92. Available from: http://www.ncbi.nlm.nih.gov/pubmed/21906798

23. O'Neil PM, Miller-Kovach K, Tuerk PW, Becker LE, Wadden TA, Fujioka K, et al. Randomized controlled trial of a nationally available weight control program tailored for adults with type 2 diabetes. Obesity. 2016;24(11):2269–77.

24. Herdman M, Gudex C, Lloyd A, Janssen M, Kind P, Parkin D, et al. Development and preliminary testing of the new five-level version of EQ-5D (EQ-5D-5L). Quality of Life Research. 2011;20(10):1727–36.

For peer review only

For peer review only - http://bmjopen.bmj.com/site/about/guidelines.xhtml

25. Devlin NJ, Shah KK, Feng Y, Mulhern B, van Hout B. Valuing health-related quality of life: An EQ-5D-5L value set for England. Health Economics (United Kingdom). 2018;27(1):7–22.

26. Al-Janabi H, Flynn TN, Coast J. Development of a self-report measure of capability wellbeing for adults: The ICECAP-A. Quality of Life Research. 2012;21(1):167–76.

27. Flynn TN, Huynh E, Peters TJ, Al-Janabi H, Clemens S, Moody A, et al. Scoring the ICECAP-A capability instrument. Estimation of a UK general population tariff. Health Economics (United Kingdom). 2015;24(3):258–69.

28. Doherty A, Jackson D, Hammerla N, Plötz T, Olivier P, Granat MH, et al. Large scale population assessment of physical activity using wrist worn accelerometers: The UK biobank study. PLoS ONE. 2017;12(2).

29. Wareham NJ, Jakes RW, Rennie KL, Mitchell J, Hennings S, Day NE. Validity and repeatability of the EPIC-Norfolk Physical Activity Questionnaire. International journal of epidemiology. 2002;31(1):168–74.

30. Golubic R, May AM, Benjaminsen Borch K, Overvad K, Charles MA, Diaz MJT, et al. Validity of electronically administered Recent Physical Activity Questionnaire (RPAQ) in ten European countries. PLoS ONE. 2014;9(3).

31. Mulligan AA, Luben RN, Bhaniani A, Parry-Smith DJ, O'Connor L, Khawaja AP, et al. A new tool for converting food frequency questionnaire data into nutrient and food group values: FETA research methods and availability. BMJ Open. 2014;4(3).

32. O'Neill J, Tabish H, Welch V, Petticrew M, Pottie K, Clarke M, et al. Applying an equity lens to interventions: Using PROGRESS ensures consideration of socially stratifying factors to illuminate inequities in health. Journal of Clinical Epidemiology. 2014;67(1):56–64.

33. Bradley C, Todd C, Gorton T, Symonds E, Martin A, Plowright R. The development of an individualized questionnaire measure of perceived impact of diabetes on quality of life: The ADDQoL. Quality of Life Research. 1999;8(1–2):79–91.

34. Dalton M, Finlayson G, Hill A, Blundell J. Preliminary validation and principal components

For peer review only

For peer review only - http://bmjopen.bmj.com/site/about/guidelines.xhtml

analysis of the Control of Eating Questionnaire (CoEQ) for the experience of food craving. European Journal of Clinical Nutrition. 2015;69(12):1313–7.

35. Westenhoefer J, Stunkard AJ, Pudel V. Validation of the flexible and rigid control dimensions of dietary restraint. International Journal of Eating Disorders. 1999;26:53–64.

36. Gormally J, Black S, Daston S, Rardin D. The assessment of binge eating severity among obese persons. Addictive Behaviors. 1982;7(1):47–55.

37. Grupski AE, Hood MM, Hall BJ, Azarbad L, Fitzpatrick SL, Corsica JA. Examining the binge eating scale in screening for binge eating disorder in bariatric surgery candidates. Obesity Surgery. 2013;23(1):1–6.

38. Griffin SJ, Borch-Johnsen K, Davies MJ, Khunti K, Rutten GE, Sandbæk A, et al. Effect of early intensive multifactorial therapy on 5-year cardiovascular outcomes in individuals with type 2 diabetes detected by screening (ADDITION-Europe): a cluster-randomised trial. The Lancet [Internet]. 2011 Jul;378(9786):156–67. Available from: http://linkinghub.elsevier.com/retrieve/pii/S0140673611606983

39. White IR, Thompson SG. Adjusting for partially missing baseline measurements in randomized trials. Statistics in Medicine. 2005;

For peer review only - http://bmjopen.bmj.com/site/about/guidelines.xhtml

Figure 1: Participant Flow Diagram

Figure 2: Schedule of Enrolment, Interventions and Assessments

For peer review only

For peer review only - http://bmjopen.bmj.com/site/about/guidelines.xhtml

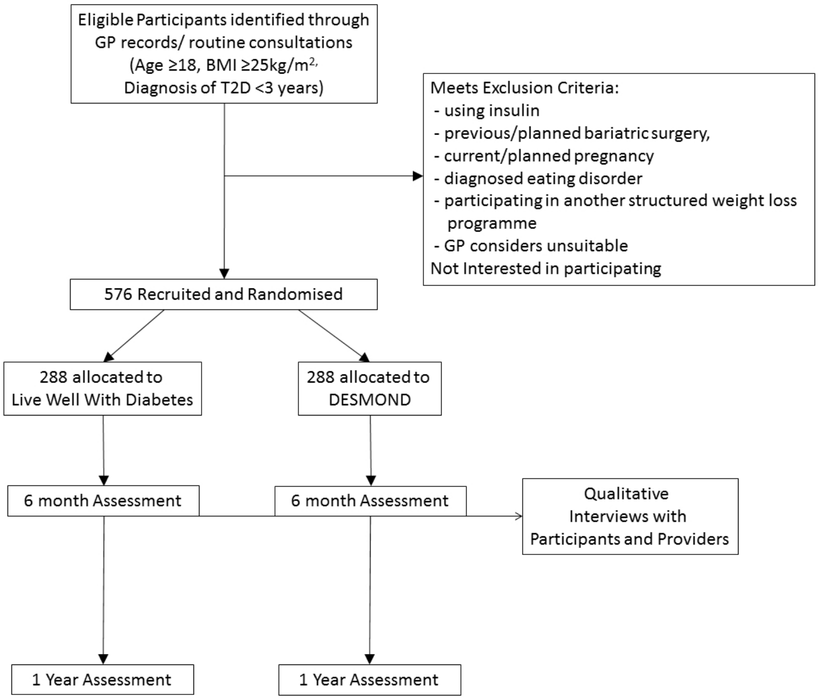

Figure 1 - Participant Flow Diagram

338x400mm (96 x 96 DPI)

	STUDY PERIOD			
	Enrolment	Baseline	Follow Up	
TIMEPOINT**	$-t_1$	0	*6 months*	*12 months*
ENROLMENT:				
Telephone Eligibility screen	X			
Informed consent		X		
Randomisation		X		
INTERVENTIONS:				
Tailored diabetes education and behavioural weight management		⟷	⟷	
Diabetes Education		X		
ASSESSMENTS:				
Height		X		
Weight		X	X	X
Body fat %		X	X	X
Blood Pressure		X	X	X
Blood sample		X	X	X
Accelerometer		X	X	X
Diet Recall		X	X	X
Self-Report Questionnaires		X	X	X
Notes Review		X	X	X
Registry Data				X

Figure 2: Schedule of Enrolment, Interventions, and Assessments

254x190mm (96 x 96 DPI)

SPIRIT

STANDARD PROTOCOL ITEMS: RECOMMENDATIONS FOR INTERVENTIONAL TRIALS

SPIRIT 2013 Checklist: Recommended items to address in a clinical trial protocol and related documents*

Section/item	Item No	Description	
**Administrative information**			
Title	1	Descriptive title identifying the study design, population, interventions, and, if applicable, trial acronym	1
Trial registration	2a	Trial identifier and registry name. If not yet registered, name of intended registry	3
	2b	All items from the World Health Organization Trial Registration Data Set	Throughout
Protocol version	3	Date and version identifier	2
Funding	4	Sources and types of financial, material, and other support	25
Roles and responsibilities	5a	Names, affiliations, and roles of protocol contributors	24
	5b	Name and contact information for the trial sponsor	ISRCTN
	5c	Role of study sponsor and funders, if any, in study design; collection, management, analysis, and interpretation of data; writing of the report; and the decision to submit the report for publication, including whether they will have ultimate authority over any of these activities	25
	5d	Composition, roles, and responsibilities of the coordinating centre, steering committee, endpoint adjudication committee, data management team, and other individuals or groups overseeing the trial, if applicable (see Item 21a for data monitoring committee)	22
**Introduction**			
Background and rationale	6a	Description of research question and justification for undertaking the trial, including summary of relevant studies (published and unpublished) examining benefits and harms for each intervention	4
	6b	Explanation for choice of comparators	4
Objectives	7	Specific objectives or hypotheses	5
Trial design	8	Description of trial design including type of trial (eg, parallel group, crossover, factorial, single group), allocation ratio, and framework (eg, superiority, equivalence, noninferiority, exploratory)	6

For peer review only

For peer review only - http://bmjopen.bmj.com/site/about/guidelines.xhtml

Methods: Participants, interventions, and outcomes

Study setting	9	Description of study settings (eg, community clinic, academic hospital) and list of countries where data will be collected. Reference to where list of study sites can be obtained	7
Eligibility criteria	10	Inclusion and exclusion criteria for participants. If applicable, eligibility criteria for study centres and individuals who will perform the interventions (eg, surgeons, psychotherapists)	7
Interventions	11a	Interventions for each group with sufficient detail to allow replication, including how and when they will be administered	8
	11b	Criteria for discontinuing or modifying allocated interventions for a given trial participant (eg, drug dose change in response to harms, participant request, or improving/worsening disease)	n/a
	11c	Strategies to improve adherence to intervention protocols, and any procedures for monitoring adherence (eg, drug tablet return, laboratory tests)	15
Outcomes	12	Primary, secondary, and other outcomes, including the specific measurement variable (eg, systolic blood pressure), analysis metric (eg, change from baseline, final value, time to event), method of aggregation (eg, median, proportion), and time point for each outcome. Explanation of the clinical relevance of chosen efficacy and harm outcomes is strongly recommended	10
Participant timeline	13	Time schedule of enrolment, interventions (including any run-ins and washouts), assessments, and visits for participants. A schematic diagram is highly recommended (see Figure)	Figure 2
Sample size	14	Estimated number of participants needed to achieve study objectives and how it was determined, including clinical and statistical assumptions supporting any sample size calculations	20
Recruitment	15	Strategies for achieving adequate participant enrolment to reach target sample size	17

Methods: Assignment of interventions (for controlled trials)

Allocation:

Sequence generation	16a	Method of generating the allocation sequence (eg, computer-generated random numbers), and list of any factors for stratification. To reduce predictability of a random sequence, details of any planned restriction (eg, blocking) should be provided in a separate document that is unavailable to those who enrol participants or assign interventions	19

Allocation concealment mechanism	16b	Mechanism of implementing the allocation sequence (eg, central telephone; sequentially numbered, opaque, sealed envelopes), describing any steps to conceal the sequence until interventions are assigned	19
Implementation	16c	Who will generate the allocation sequence, who will enrol participants, and who will assign participants to interventions	19
Blinding (masking)	17a	Who will be blinded after assignment to interventions (eg, trial participants, care providers, outcome assessors, data analysts), and how	19
	17b	If blinded, circumstances under which unblinding is permissible, and procedure for revealing a participant's allocated intervention during the trial	19
**Methods: Data collection, management, and analysis**			
Data collection methods	18a	Plans for assessment and collection of outcome, baseline, and other trial data, including any related processes to promote data quality (eg, duplicate measurements, training of assessors) and a description of study instruments (eg, questionnaires, laboratory tests) along with their reliability and validity, if known. Reference to where data collection forms can be found, if not in the protocol	12
	18b	Plans to promote participant retention and complete follow-up, including list of any outcome data to be collected for participants who discontinue or deviate from intervention protocols	12
Data management	19	Plans for data entry, coding, security, and storage, including any related processes to promote data quality (eg, double data entry; range checks for data values). Reference to where details of data management procedures can be found, if not in the protocol	24
Statistical methods	20a	Statistical methods for analysing primary and secondary outcomes. Reference to where other details of the statistical analysis plan can be found, if not in the protocol	20
	20b	Methods for any additional analyses (eg, subgroup and adjusted analyses)	20-22
	20c	Definition of analysis population relating to protocol non-adherence (eg, as randomised analysis), and any statistical methods to handle missing data (eg, multiple imputation)	20
**Methods: Monitoring**			
Data monitoring	21a	Composition of data monitoring committee (DMC); summary of its role and reporting structure; statement of whether it is independent from the sponsor and competing interests; and reference to where further details about its charter can be found, if not in the protocol. Alternatively, an explanation of why a DMC is not needed	22

	21b	Description of any interim analyses and stopping guidelines, including who will have access to these interim results and make the final decision to terminate the trial	23
Harms	22	Plans for collecting, assessing, reporting, and managing solicited and spontaneously reported adverse events and other unintended effects of trial interventions or trial conduct	23
Auditing	23	Frequency and procedures for auditing trial conduct, if any, and whether the process will be independent from investigators and the sponsor	23
**Ethics and dissemination**			
Research ethics approval	24	Plans for seeking research ethics committee/institutional review board (REC/IRB) approval	24
Protocol amendments	25	Plans for communicating important protocol modifications (eg, changes to eligibility criteria, outcomes, analyses) to relevant parties (eg, investigators, REC/IRBs, trial participants, trial registries, journals, regulators)	24
Consent or assent	26a	Who will obtain informed consent or assent from potential trial participants or authorised surrogates, and how (see Item 32)	18
	26b	Additional consent provisions for collection and use of participant data and biological specimens in ancillary studies, if applicable	n/a
Confidentiality	27	How personal information about potential and enrolled participants will be collected, shared, and maintained in order to protect confidentiality before, during, and after the trial	24
Declaration of interests	28	Financial and other competing interests for principal investigators for the overall trial and each study site	25
Access to data	29	Statement of who will have access to the final trial dataset, and disclosure of contractual agreements that limit such access for investigators	26
Ancillary and post-trial care	30	Provisions, if any, for ancillary and post-trial care, and for compensation to those who suffer harm from trial participation	n/a
Dissemination policy	31a	Plans for investigators and sponsor to communicate trial results to participants, healthcare professionals, the public, and other relevant groups (eg, via publication, reporting in results databases, or other data sharing arrangements), including any publication restrictions	24
	31b	Authorship eligibility guidelines and any intended use of professional writers	24
	31c	Plans, if any, for granting public access to the full protocol, participant-level dataset, and statistical code	26

For peer review only - http://bmjopen.bmj.com/site/about/guidelines.xhtml

Appendices

Informed consent materials	32	Model consent form and other related documentation given to participants and authorised surrogates	Supplied to HRA
Biological specimens	33	Plans for collection, laboratory evaluation, and storage of biological specimens for genetic or molecular analysis in the current trial and for future use in ancillary studies, if applicable	Supplied to HRA

*It is strongly recommended that this checklist be read in conjunction with the SPIRIT 2013 Explanation & Elaboration for important clarification on the items. Amendments to the protocol should be tracked and dated. The SPIRIT checklist is copyrighted by the SPIRIT Group under the Creative Commons "Attribution-NonCommercial-NoDerivs 3.0 Unported" license.

For peer review only

BMJ Open

The clinical and cost-effectiveness of a diabetes education and behavioural weight management programme versus a diabetes education programme in adults with a recent diagnosis of type 2 diabetes: Study protocol for the Glucose Lowering through Weight Management (GLoW) randomised controlled trial.

Journal:	*BMJ Open*
Manuscript ID	bmjopen-2019-035020.R1
Article Type:	Protocol
Date Submitted by the Author:	29-Jan-2020
Complete List of Authors:	Ahern, Amy L.; University of Cambridge School of Clinical Medicine, MRC Epidemiology Unit<br>Woolston, Jenny; University of Cambridge School of Clinical Medicine, MRC Epidemiology Unit<br>Wells, Emma; University of Cambridge School of Clinical Medicine, MRC Epidemiology Unit<br>Sharp, Stephen; University of Cambridge School of Clinical Medicine, MRC Epidemiology Unit<br>Islam, Nazrul; University of Cambridge School of Clinical Medicine, MRC Epidemiology Unit<br>Lawlor, Emma; University of Cambridge School of Clinical Medicine, MRC Epidemiology Unit<br>Duschinsky, Robbie; University of Cambridge Primary Care Unit<br>Hill, Andrew; University of Leeds, School of Medicine<br>Doble, Brett; Duke-NUS Medical School<br>Wilson, Ed; University of East Anglia, Norwich Medical School<br>Morris, Stephen; University of Cambridge School of Clinical Medicine, Primary Care Unit<br>Hughes, Carly; Fakenham Medical Practice; University of East Anglia Faculty of Medicine and Health Sciences<br>Brennan, Alan; The University of Sheffield, School of Health and Related Research<br>Bostock, Jennifer; Patient and Public Involvement Representative<br>Boothby, Clare; University of Cambridge School of Clinical Medicine, MRC Epidemiology Unit<br>Griffin, Simon; University of Cambridge School of Clinical Medicine, MRC Epidemiology Unit; University of Cambridge School of Clinical Medicine, Primary Care Unit, Institute of Public Health
**Primary Subject Heading**:	Diabetes and endocrinology
Secondary Subject Heading:	General practice / Family practice
Keywords:	Diabetes & endocrinology < INTERNAL MEDICINE, PRIMARY CARE, General diabetes < DIABETES & ENDOCRINOLOGY, GENERAL MEDICINE

	(see Internal Medicine)

SCHOLARONE™
Manuscripts

I, the Submitting Author has the right to grant and does grant on behalf of all authors of the Work (as defined in the below author licence), an exclusive licence and/or a non-exclusive licence for contributions from authors who are: i) UK Crown employees; ii) where BMJ has agreed a CC-BY licence shall apply, and/or iii) in accordance with the terms applicable for US Federal Government officers or employees acting as part of their official duties; on a worldwide, perpetual, irrevocable, royalty-free basis to BMJ Publishing Group Ltd ("BMJ") its licensees and where the relevant Journal is co-owned by BMJ to the co-owners of the Journal, to publish the Work in this journal and any other BMJ products and to exploit all rights, as set out in our licence.

The Submitting Author accepts and understands that any supply made under these terms is made by BMJ to the Submitting Author unless you are acting as an employee on behalf of your employer or a postgraduate student of an affiliated institution which is paying any applicable article publishing charge ("APC") for Open Access articles. Where the Submitting Author wishes to make the Work available on an Open Access basis (and intends to pay the relevant APC), the terms of reuse of such Open Access shall be governed by a Creative Commons licence – details of these licences and which Creative Commons licence will apply to this Work are set out in our licence referred to above.

Other than as permitted in any relevant BMJ Author's Self Archiving Policies, I confirm this Work has not been accepted for publication elsewhere, is not being considered for publication elsewhere and does not duplicate material already published. I confirm all authors consent to publication of this Work and authorise the granting of this licence.

For peer review only

V4 16.09..2019

The clinical and cost-effectiveness of a diabetes education and behavioural weight management programme versus a diabetes education programme in adults with a recent diagnosis of type 2 diabetes: Study protocol for the Glucose Lowering through Weight Management (GLoW) randomised controlled trial.

Dr Amy L. Ahern[1*], Mrs Jenny Woolston[1], Ms Emma Wells[1], Mr Stephen J. Sharp[1], Dr Nazrul Islam[1], Dr Emma Lawlor[1], Dr Robbie Duschinsky[2], Professor Andrew J. Hill[3], Dr Brett Doble[4], Dr Ed Wilson[5], Professor Stephen Morris[2], Dr Carly A. Hughes [5,6], Professor Alan Brennan[7], Mrs Jennifer Bostock[8], Dr Clare E. Boothby[1], Professor Simon J. Griffin[1,2]

1. MRC Epidemiology Unit, Box 285 Institute of Metabolic Sciences, School of Clinical Medicine, University of Cambridge, Cambridge, CB2 0QQ, UK
2. The Primary Care Unit, Institute of Public Health, School of Clinical Medicine, University of Cambridge, Forvie Site, Cambridge, CB2 OSR, UK
3. School of Medicine, University of Leeds, Worsley Building, Leeds, LS2 9JT, UK
4. Duke-NUS Medical School, College Road, Singapore 169857
5. Norwich Medical School, University of East Anglia, Norwich Research Park, Norwich, NR4 7TJ, UK
6. The Fakenham Medical Practice, Fakenham, UK
7. School of Health and Related Research, University of Sheffield, Regent Court, Regent Street, Sheffield, SD1 4DA, UK
8. Patient and Public Involvement Representative

* Corresponding author: MRC Epidemiology Unit, University of Cambridge, Box 285 Institute of Metabolic Science, Cambridge Biomedical Campus, Cambridge, UK, CB2 0QQ, ala34@cam.ac.uk

For peer review only

For peer review only - http://bmjopen.bmj.com/site/about/guidelines.xhtml

For peer review only

For peer review only - http://bmjopen.bmj.com/site/about/guidelines.xhtml

Abstract

Introduction: People with type 2 diabetes can improve glycaemic control or even achieve remission through weight loss, and reduce their use of medication and risk of cardiovascular disease. The Glucose Lowering through Weight management (GLoW) trial will evaluate whether a tailored diabetes education and behavioural weight management programme is more effective and cost-effective than a diabetes education programme in helping people with overweight or obesity and a recent diagnosis of type 2 diabetes to lower their blood glucose, lose weight, and improve other markers of cardiovascular risk.

Methods and Analysis: Pragmatic, randomised, single-blind, parallel group, two-arm, superiority trial. We will recruit 576 adults with BMI>25kg/m^2 and diagnosis of type 2 diabetes in the past 3 years, and randomise them to a tailored diabetes education and behavioural weight management programme or a diabetes education programme. Participants will attend measurement appointments at a local GP practice or research centre at baseline, 6 and 12 months. The primary outcome is 12 month change in glycated haemoglobin. The effect of the intervention on the primary outcome will be estimated and tested using a linear regression model (ANCOVA) including randomisation group and adjusted for baseline value of the outcome and the randomisation stratifiers. Participants will be included in the group to which they were randomised, under the intention-to-treat principle. Secondary outcomes include 6 and 12 month changes in body weight, body fat percentage, systolic and diastolic blood pressure, and lipid profile; probability of achieving good glycaemic control; probability of achieving remission from diabetes; probability of losing 5% and 10% body weight; and modelled cardiovascular risk (UKPDS). An intention-to-treat within-trial cost effectiveness analysis will be conducted from NHS and societal perspectives using participant-level data. Qualitative interviews will be conducted with participants to understand why and how the programme achieved its results and how participants manage their weight after the programme ends.

Ethics and Dissemination: Ethical approval was received from East of Scotland Research Ethics Service on 15th May 2018 (18/ES/0048). This protocol (version 3) was approved on 19th June 2019.

For peer review only - http://bmjopen.bmj.com/site/about/guidelines.xhtml

Findings will be published in peer-reviewed scientific journals and communicated to other stakeholders as appropriate.

Trial registration: ISRCTN18399564, registered 9th July 2018

Keywords

Weight management, type 2 diabetes, diabetes education, primary care, randomised controlled trial

Strengths and Limitations

- This trial will provide robust evidence of the clinical and cost-effectiveness of a scalable tailored diabetes education and behavioural weight management programme versus diabetes education alone in adults with a recent diagnosis of type 2 diabetes.
- If shown to be cost-effective, the intervention being evaluated could be readily integrated into existing UK care pathways and delivered to large numbers of people.
- The behavioural programme is already widely available across many countries and this model of care could be readily adopted across health care services internationally.
- The trial only includes follow up at 6 months and 12 months and longer term data may be needed to understand the longer term impact of these programmes.
- Consent has been obtained to follow up participants through national registries and medical records for up to 15 years.

For peer review only

For peer review only - http://bmjopen.bmj.com/site/about/guidelines.xhtml

Background

The treatment of diabetes and related complications (e.g. cardiovascular disease, amputation, kidney failure) uses approximately10% of the UK NHS budget.(1) This is predicted to rise to 17% in 2035 as the number of people with diabetes in the UK rises to 6.25 million, of which 5.6 million cases will be adults with type 2 diabetes (T2D).(1) Adults who are living with T2D are at increased risk of developing physical and mental health comorbidities, and have reduced quality of life and shorter life expectancy.(2,3) There are considerable social and economic costs to the individual living with diabetes, as well as to wider society.(1,2,4)

While T2D is typically characterised as a progressive, irreversible condition, there is evidence of remission in patients losing weight through bariatric surgery(5,6) or closely supervised very-low-calorie formula diets.(7)·(8) However, many patients with T2D may be unsuitable for or unwilling to undergo these interventions and, given their high cost and reliance on specialists, they are unlikely to be widely adopted in the NHS in the near future. Partial or complete remission of type 2 diabetes has also been observed following smaller weight losses achieved through behavioural interventions.(9) Moreover, even without remission, weight loss and behaviour change can produce improvements in health outcomes for people who have diabetes. We have shown that losing a moderate amount of weight or making healthy behaviour changes (e.g. increasing physical activity, reducing alcohol, energy and fat intake) in the first year after diagnosis can reduce the likelihood of stroke or heart attack in the next 5-10 years(10–12) and increase the likelihood of achieving remission at 5 years.(13)

The Look AHEAD trial demonstrated that intensive specialist-led behavioural programmes could lead to weight loss and reductions in cardiovascular risk factors over 8 year follow up.(14) However, there are currently insufficient resources in the UK NHS to provide intensive, specialist-led behavioural programmes to the 3.2 million individuals who have T2D and the additional 200,000 who are diagnosed each year. Instead, current guidelines focus on structured diabetes education and dietary advice,(15) which is cheaper and scalable but has small, short-term effects on weight and glycaemia, and relatively poor uptake.(16–18) A recent systematic review found that supportive behaviour-change

For peer review only

For peer review only - http://bmjopen.bmj.com/site/about/guidelines.xhtml

programmes (with ≥11 hours of contact time) achieve greater reductions in weight and HbA_{1c} than structured education without additional support (≤10hrs).(17) Integrating effective but scalable behaviour change programmes into care pathways for T2D could potentially improve patients' glycaemic control and related risk factors and reduce complications. This would improve health and quality of life for people living with diabetes and reduce the burden of diabetes on health care resources.

We have previously shown that commercial open-group behavioural weight management programmes, such as WW (formerly Weight Watchers®) or Slimming World, offer a scalable and cost-effective way to help people lose weight and reduce risk of diabetes.(19–22) A randomised trial in the US showed that a combination of WW classes and remote dietary counselling achieved greater weight losses and reductions in HbA_{1c} than standard care over 1 year in people with diabetes.(23) A quarter of participants randomised to this programme achieved good glycaemic control (HbA_{1c} below 53mmol/mol) at 12 months, compared with 14% of those receiving standard care. In the UK, a similar intervention has been developed for use in the NHS that combines referral to WW with NICE-compliant diabetes education and dietary advice. However, this programme is unlikely to be widely commissioned without robust evidence of cost-effectiveness. The proposed trial will provide reliable evidence on the relative effectiveness and cost-effectiveness of a tailored diabetes education and behavioural weight management programme vs diabetes education, for people with a recent diagnosis of T2D (≤3years).

Objectives

Primary Objective

To evaluate the effect of a tailored diabetes education and behavioural weight management programme (DEW) vs diabetes education (DE) on glycated haemoglobin (HbA_{1c}) at 12 months in adults with a recent diagnosis of type 2 diabetes.

For peer review only - http://bmjopen.bmj.com/site/about/guidelines.xhtml

Secondary Objectives

To evaluate the effect of DEW vs DE on:

- body weight, body fat percentage, blood pressure, lipid profile, and modelled 10 year cardiovascular risk at 6 and 12 months
- probability of achieving clinically significant weight loss, good glycaemic control or diabetes remission at 6 and 12 months
- changes in diet and physical activity at 6 and 12 months
- psychosocial factors associated with successful weight control at 6 and 12 months.

To evaluate the within-trial cost-effectiveness of DEW vs DE.

To assess the extent to which the two programmes reach the target population.

To explore participant and practitioner experiences of the two programmes and the extent to which these programmes meet their needs.

To clarify causal mechanisms, and identify contextual factors associated with variations in outcome.

To identify barriers and facilitators to maintenance of weight management behaviours after treatment cessation.

Methods and Analysis

Trial Design

This is a pragmatic, randomised, single-blind, parallel group, two-arm, superiority trial. Participants will be randomised to either the tailored diabetes education and behavioural weight management programme or to diabetes education. Block randomisation will be used with a 1:1 allocation stratified by sex and duration of diabetes (Figure 1).

For peer review only - http://bmjopen.bmj.com/site/about/guidelines.xhtml

Study Setting

Research active primary care practices in the East of England that currently refer patients with type 2 diabetes (≤3 years duration) to local structured diabetes education (DESMOND) and have active WW groups in the local community.

Participants

Participants will be 576 adults with overweight or obesity who have a diagnosis of type 2 diabetes in the past 3 years.

Inclusion criteria

- BMI ≥25kg/m^2
- Age ≥18 years
- Diagnosis of type 2 diabetes within the previous 36 months (confirmatory blood test will not be required)
- Capable of giving informed consent
- Have a good understanding of the English language (study materials are not tailored to support non-English language speakers)
- Willing to be randomised
- Willing to attend follow up visits at a local participating GP practice or research centre

Exclusion criteria

- Using insulin
- Previous/planned bariatric surgery
- Current/planned pregnancy
- Current diagnosis of eating disorder
- Already received a structured diabetes education programme

For peer review only - http://bmjopen.bmj.com/site/about/guidelines.xhtml

- GP considers unsuitable
- Participation in another structured behaviour change programme for diet and/or physical activity within the past 3 months

Interventions

Tailored diabetes education and behavioural weight management programme

The tailored structured education and behavioural weight management programme was developed and is delivered by WW. It lasts 6 months and is overseen by a registered dietitian with experience in diabetes, diet and behaviour counselling and specific training in the standard WW programme.

Structured diabetes education

The structured diabetes education programme is delivered remotely (video conferencing /telephone) on a 1:1 basis by the registered dietitian over two education sessions, 3-5 weeks apart (total time 1 hr 30 minutes, divided between 2 calls). It covers a standardised QISMET-accredited core curriculum but can then be adapted for the individual's needs. Additional self-help education materials to support the curriculum will be available online and are delivered to all participants via email or mail, at the patient's preference. These materials may be minimally tailored to the individual participant. Information on the materials can be found at www.weightwatchers.com/uk/live-well-diabetes. During the education sessions, materials on goal setting, understanding diabetes, the glycaemic index, carbohydrates, physical activity, and weight management are reviewed.

Behavioural programme

The behavioural weight management component consists of free membership of WW for 6 months. This includes attendance at weekly in-person group meetings (30-40 minutes) held at a variety of times in a range of local community venues. These are open-group meetings (new people may join or leave the group at any time) and are led by a coach (trained lay person with experience of the programme). Meetings include a confidential weigh in with the coach and a 30-minute interactive

For peer review only - http://bmjopen.bmj.com/site/about/guidelines.xhtml

education session led by the coach that includes advice on diet, physical activity, and positive mindset, using behavioural strategies (e.g. goal setting, self-monitoring, problem solving, modifying the personal food environment, and relapse prevention). Peer support is available from coaches and other group members. Participants can be accompanied by a friend, relative or carer. Participants will also have access to the WW app, online digital tools, and standard materials such as recipe booklets, physical activity guidance and meal trackers for the duration of the intervention. Participants can contact their coach for support/advice between meetings via an online chat function.

Participant engagement

Once a referral is received, the registered dietitian will telephone the participant, welcome them to the programme, sign them up to a local WW meeting, and arrange the first education session. Participants are also given an information sheet with details of how to contact the dietitian directly. A closed social media support group will be formed and all participants will be encouraged to join if they wish to do so. Activity on the group forum will be monitored and supported by the registered dietitian. Each week, the dietitian will contact 2-5 participants (selected at random from those who have participated in the programme for longer than 3 months) via the social media group or phone. Participants can also contact the dietitian proactively during the intervention period for additional remote support where needed, but will be encouraged to do so via the social media group in the first instance. If participants miss 4 or more WW standard in-person meetings their local coach will call to help the individual return to the programme.

Diabetes Education

In the East of England, the diabetes education programme most commonly commissioned by the Clinical Commissioning Group (CCG) is the Diabetes and Education Self-Monitoring for Ongoing and Newly Diagnosed (DESMOND) programme.(16,18) This is a structured diabetes education programme for people with a recent diagnosis of type 2 diabetes (≤3 years since diagnosis).

For peer review only - http://bmjopen.bmj.com/site/about/guidelines.xhtml

Participants can attend 6 hours of structured self-management group education, covering: thoughts and feelings about diabetes; understanding diabetes and glucose - what happens in the body; understanding risk factors and complications associated with diabetes; understanding monitoring and medication; how to take control - food choices and physical activity; and planning for the future. The structured education is delivered in 1 day or 2 half days by two trained health care professionals in local health care or community venues. Sessions are delivered in groups of up to 10 participants, and participants can bring a friend or partner with them. The education sessions are supported by specially developed resources.

Outcomes

Primary Outcome

12 month change from baseline in glycated haemoglobin (HbA_{1c}).

Secondary Outcomes

6 month change from baseline in HbA_{1c}.

6 and 12 month changes from baseline in body weight, body fat percentage, systolic and diastolic blood pressure, total cholesterol, HDL cholesterol, and LDL cholesterol.

Good glycaemic control (HbA_{1c} <53mmol/mol) at 6 and 12 months.

Remission from diabetes (HbA_{1c} <48mmol/mol and without glucose-lowering medication for ≥2 months) at 6 and 12 months.

Weight loss ≥5% and ≥10% of initial body weight at 6 and 12 months.

Modelled cardiovascular risk (UKPDS) at 12 months.

For peer review only - http://bmjopen.bmj.com/site/about/guidelines.xhtml

Behavioural and Psychosocial Outcomes

6 and 12 month changes from baseline in objectively measured physical activity (accelerometry), self-reported physical activity, objective marker of fruit and vegetable intake (plasma carotenoids), and self-reported dietary intake.

6 and 12 months changes from baseline, adjusted for baseline, in dietary restraint, control over food cravings, emotional eating, self-regulatory skills, social support and diabetes related quality of life.

Health Economic Outcomes

Detailed micro-costing of DEW and DE.

Health and social care resource use over 12 months (medical notes, registry data, resource use questionnaire).

Self-reported out-of-pocket costs and lost productivity (e.g. due to days off work, up to 12 months).

12 month quality-adjusted life-years (QALYs) based on Health Related Quality of Life (HRQoL) (EQ-5D-5L)(24,25) and capability/wellbeing (ICECAP-A)(26,27)

Total and incremental costs from NHS and societal perspectives; incremental net (monetary) benefit; incremental cost-effectiveness and cost-utility ratios; value of information estimates.

Uptake and adherence

Number and characteristics of participants who:

- are offered the opportunity to participate in the trial
- enrol in the trial
- attend the intervention
- adhere to the intervention (including specific intervention components)

For peer review only

A detailed protocol for process evaluation will also be developed.

Visits and Measurements

Visit Schedule

Participants will be asked to attend measurement appointments at a participating primary care practice or research centre at 0, 6, and 12 months. Details of measures at each assessment are summarised in Figure 2. Participants will be reimbursed for reasonable travel expenses and given an honorarium for attending measurement appointments (£10 for baseline and 6 month visits, £30 for the 12 month visit). Honoraria for assessment attendance are not dependent on intervention attendance/completion.

Anthropometric Measures

Anthropometric measurements will be made at participating primary care practices or research centres by research-trained health care professionals (hereafter "research nurses") blind to intervention allocation, in line with standardised operating procedures. Participants will be asked to remove shoes and heavy clothing items. Height will be measured in cm using a mounted stadiometer (make and model dependent on practice). Where possible, weight (kg) and body fat percentage will be measured using a calibrated Tanita segmental body composition analyser (Tanita Ltd; MA Tokyo, Japan; model dependent on practice), which will be provided to practices by the research team. Where we have an insufficient number of Tanita scales to measure body fat at a practice, we will measure weight (kg) only, using calibrated electronic scales (model dependent on practice). To maximise the number of participants for whom we can measure body fat, we will prioritise the use of Tanitas at large practices with a large number of eligible patients, and CRN-led practices (as CRN nurses can transport the Tanita scales to numerous practices). Blood pressure will be measured 3 times in a resting state using a calibrated OMRON automatic blood pressure monitor (OMRON Healthcare UK Ltd: Milton Keynes, UK; model dependent on practice).

For peer review only - http://bmjopen.bmj.com/site/about/guidelines.xhtml

If a research nurse is not available to conduct follow up assessments, assessments may be conducted by trained research centre staff. If participants are unable to attend follow up appointments at a participating practice, the research team may offer appointments at the local hospital or research centre, or home visits. Participants who are unable or unwilling to attend a visit will be asked to provide a self-measured weight. All other participants will also be asked for a self-measured weight at the time of appointment booking, to enable us to quantify the degree of misreporting of self-measured weights.

Biochemical Measures

Participants will be asked to provide a blood sample for the measurement of HbA_{1c}, lipid profile, and carotenoids. If possible, this will be taken at the practice at the same time as the anthropometric measurements. Blood samples will be collected in three tubes labelled with an anonymised barcode (4.9ml serum tube for lipid profile, 2.6ml EDTA tube for $HbA_{1c,}$ 4.9ml lithium heparin (LH) tube for carotenoids). The LH tube is light sensitive, so will be wrapped in foil to avoid degradation. Samples will be placed in a Royal Mail Safebox™, and posted via first class mail to the central laboratory for analysis. Each sample is stable for up to 3 days at room temperature. Plasma samples for analysis of carotenoids will be frozen and analysed in batches.

Behavioural Measures

Physical Activity

Physical activity will be measured objectively using a triaxial accelerometer (Axivity AX3, Newcastle, UK) which measures raw acceleration (m/s^2). Participants will be asked to wear the accelerometer on their non-dominant wrist continuously for 7 consecutive days and nights. The device is small, light and waterproof and is worn like a watch. It does not need to be taken off if showering, bathing or swimming.

For peer review only - http://bmjopen.bmj.com/site/about/guidelines.xhtml

At the baseline visit, the nurse will take the participant through the accelerometer instruction sheet. They will demonstrate to the participant how they should wear the monitor on their wrist. The nurse will also ensure that the timing of the monitor and return is feasible for the participant. The study team will initialise the accelerometer and will post it out to the participant with the instruction sheet. The participant will wear the monitor for 7 days and nights and will complete an activity log. For the 6 and 12 month visits, accelerometers will be mailed out to participants. At the end of 7 days the participant will be asked to post the device back to the research centre using pre-paid, pre-addressed materials. Trained staff will download the data from the accelerometer and check it to ensure the integrity of the data recording. This method has been used in large-scale epidemiological studies including the UK Biobank study in 100,000 individuals.(28) Data will be analysed using established methods used in UK Biobank. The outcome variables of interest are vector magnitude, a measure of total physical activity, and intensity distribution, from which duration of time in different levels of physical activity can be inferred.

Self-reported physical activity will be measured using the Recent Physical Activity Questionnaire (RPAQ), a validated measure of four domains of physical activity (leisure time, occupation, commuting, and domestic life) that has been used in a number of intervention studies and large-scale epidemiological studies. (29,30) This will be administered as part of the self-report questionnaires.

Diet

Plasma carotenoids will be measured as an objective marker of fruit and vegetable intake. Self-reported dietary intake will be measured at each timepoint using a version of the previously validated EPIC Food Frequency Questionnaire (FFQ)(31) that has been adapted for a 6 month recall period. This will be administered as part of the self-report questionnaires

Self-report Questionnaires

Participants will complete a demographics questionnaire at baseline based on Progress-Plus(32) factors (place of residency, race/ethnicity, occupation, gender/sex, religion, education, socioeconomic

For peer review only - http://bmjopen.bmj.com/site/about/guidelines.xhtml

status, social capital, age, disability, relationship status, caring responsibilities, car ownership, access to the internet).

Self-reported behavioural and psychosocial measures will be collected via validated self-report questionnaires, which can be completed on paper or online, at the participant's preference. A full list of questionnaires can be found in Table 1.

Intervention Adherence

Adherence data for the tailored diabetes education and behavioural weight management programme (including attendance at weekly WW meetings and remote education sessions, and use of online tools) will be collected by WW as part of an established system for NHS contracts. Self-reported intervention adherence for both programmes will also be collected via a self-report questionnaire.

Table 1: Questionnaires administered in the GLoW trial

**Domain**	**Measure**	**Time point**
Demographics	Bespoke questionnaire based on PROGRESS-Plus(32)	Baseline
Health-related quality of life	EQ-5D-5L(24,25)	All
Capability/Wellbeing	ICECAP-A(26,27)	All
Diabetes-related quality of life	Audit of Diabetes Dependent Quality of Life - ADDQoL(33)	All
Health/ Social Care Use	Bespoke resource use questionnaire	All
Food Cravings	Control of Eating Questionnaire(34)	All
Dietary Restraint	Three Factor Eating Questionnaire – Restraint subscale(35)	All
Binge Eating	The Binge Eating Scale (BES)(36,37)	All
Dietary Intake	Food Frequency Questionnaire(31)	All

For peer review only

For peer review only - http://bmjopen.bmj.com/site/about/guidelines.xhtml

Physical Activity	Recent Physical Activity Questionnaire (RPAQ)(29,30)	All
Intervention adherence	Bespoke questionnaire	6 months

Medical Notes Review

Healthcare resource use will be obtained for all participants via medical notes review and registry data. Primary care records will be used to extract numbers of visits to the GP and community health care workers (defined as any primary or community based health worker such as nurse and allied health professional contacts noted in the patient's record) and prescribed medications. Last recorded weight, HbA_{1c} value, smoking status and diabetes status will also be extracted. This will help to minimise missing data from missed assessment appointments.

The notes review will be carried out at each study visit and following the end of the study by a research nurse blind to intervention allocation.

- Baseline visit: notes review to cover the 3 months prior to study start
- 6 month visit: notes review to cover from baseline up to 6 months
- 12 month visit: notes review to cover from 6 months to 12 months

We will obtain consent to conduct future notes reviews after the study has completed (up to 15 years post study) for future studies of long term outcomes.

Administrative and Registry Data

We will obtain consent at baseline for future collection of administrative and registry data on health event and health care usage information (up to 15 years post-study). Outcomes will include hospital episode statistics, cardiovascular events, stroke, myocardial infarction, cancer and death, and will be obtained from NHS digital, the Healthcare Quality Improvement Partnership (HQIP) and the National Cancer Registration and Analysis Service (NCRAS). This will allow us to evaluate the longer term impact of these programmes on diabetes- and weight-related morbidity and mortality.

For peer review only

Qualitative Interviews

Around the time of the 6 month follow up, we will conduct semi-structured interviews with participants from both intervention arms (N ≥ 26). Participants will be recruited in a 2:1 ratio (DEW:DE), as we are interested in comparing the experience of the two groups, but we expect the group receiving the tailored programme to provide the richest data on the experiences of weight loss and weight loss maintenance. Participants will be purposively sampled (demographic characteristics, programme attendance/adherence, weight change). We will also conduct interviews with treatment providers (N ≥ 12) to understand their experience of delivering the intervention. Interviews will be conducted by a trained research associate and audio recorded.

Questions will focus on exploring:

- How did participants and practitioners experience the programme and its components: i) education; ii) WW meetings; iii) digital tools; iv) remote dietetic counselling?
- What were the causal mechanisms and contextual factors that were associated with different outcomes?
- What were the needs of participants at the end of the programme?
- What were the facilitators and barriers to maintaining behavioural change?

Recruitment and Enrolment

Recruitment of Practices

Primary care practices will be identified and recruited by Clinical Research Networks (CRN). Participating practices must be currently referring patients with a recent diagnosis of type 2 diabetes to structured diabetes education (DESMOND) as part of standard practice and should have active WW meetings in the local area. They should also be research active with the capacity to recruit and follow up participants. The CRN will attempt to recruit practices from diverse areas using known characteristics of the practice population, to enable recruitment of a sample broadly representative of

For peer review only - http://bmjopen.bmj.com/site/about/guidelines.xhtml

the target population (UK adults with overweight and obesity who have a recent diagnosis of type 2 diabetes).

Patient identification

Eligible patients will be identified through electronic searches of primary care records and waiting lists for diabetes education. GPs will write to all potentially eligible patients inviting them to participate. This letter will include a participant information sheet and details of how to contact the study team at the research centre. Participants will also receive opportunistic invitations during routine consultations, via an invitation letter and participant information sheet to take home with them. Pop up alerts on patient records (triggered by diagnosis or attempted referral to diabetes education) will be implemented to facilitate opportunistic recruitment. To boost enrolment, advertisements will also be placed in local pharmacies, news media and other relevant settings.

Enrolment

Patients willing to participate will be asked to contact the research centre for more information. This can be by telephone, email, or reply slip. Research centre staff will answer any questions and conduct a telephone screening. If patients are eligible and willing to participate, the research staff will check that they have received the participant information sheet (sending another if they have not) and arrange a baseline assessment with the research nurse at a local participating practice or research centre.

At the baseline visit, the research nurse will take informed consent. This will include ensuring that participants have read the information sheet and had the opportunity to ask questions. They will also confirm eligibility, including an objective measure of height and weight, before taking the rest of the baseline measurements. At the end of each day, the research nurse will email the study coordinator a list of participants who have formally enrolled on the trial.

For peer review only - http://bmjopen.bmj.com/site/about/guidelines.xhtml

Randomisation

Randomisation sequence

Participants will be allocated to one of the two intervention arms in a 1:1 allocation using individual-level blocked randomisation stratified by sex (male, female) and duration of diabetes (<1 year, 1-3 years) with a block size of 6. The randomisation sequence will be computer-generated by the trial statistician and the randomisation process implemented by the data manager. The sequence will be unknown to all other personnel, including study coordinators, outcome assessors and investigators.

Method of implementing the allocation sequence

When participant eligibility has been confirmed at the baseline visit, the practice will inform the study coordinator. The study coordinator will enter the participant's details into the trial database, which will automatically assign an intervention to the participant.

The study coordinator (or member of their team) will write to the participant to inform them of their intervention allocation, and will also inform the participant's GP. The study coordinator or the GP (depending on practice and intervention allocation) will also send a referral form to the intervention provider, giving the details of the participant who has been referred to them.

Blinding

Given the nature of the intervention, it is not possible to blind participants to their intervention group. GPs will be informed of the intervention allocation and participants will be allowed to discuss the intervention with their GP, as they would outside a trial scenario. However, participants will be asked not to reveal their intervention group to outcome assessors (i.e. research nurses taking measurements). The trial statistician and the Investigators will be blinded to intervention allocation until the database is locked and the primary analysis complete.

For peer review only

For peer review only - http://bmjopen.bmj.com/site/about/guidelines.xhtml

Statistics and Data Analysis

Sample size calculation

The primary outcome is 12 month change from baseline in HbA_{1c}. Based on data from a previous trial in adults with a recent diagnosis of T2D,(38) we assumed a 16mmol/mol SD, a 0.8 correlation between baseline and follow-up and 25% attrition. In a US trial of a similar intervention in people with T2D of any duration, a difference of 4mmol/mol was observed between intervention (-3mmol/mol) and control (+1mml/mol) at 12 months.(23) We need 576 participants to detect a difference between randomised groups of 3mmol/mol HbA_{1c} with 90% power at a 5% significance level.

Statistical analysis plan

A detailed statistical analysis plan will be developed and signed off by the Trial Steering Committee prior to analysis. Participants will be analysed in the group to which they were randomised, based on the intention-to-treat principle. The intervention effect, representing the baseline-adjusted difference in change from baseline to 12 months in HbA_{1c} between the intervention and control group, will be estimated using a linear regression model including randomisation group, baseline value of HbA_{1c} (i.e. analysis of covariance, ANCOVA), and the randomisation stratifiers (sex, duration of diabetes). The missing indicator method(39) will be used to ensure inclusion of participants with a missing baseline value of HbA_{1c}. Participants with missing values of HbA_{1c} at 12 months will be excluded (i.e. a complete-case analysis). If there are >5% of participants with missing values of HbA_{1c} at 12 months, a sensitivity analysis will be performed using multiple imputation by chained equations; full details of this analysis will be provided in the statistical analysis plan.

Continuous secondary outcomes will be analysed using the method described above. Binary secondary outcomes will be analysed using a logistic regression model including randomisation group and the randomisation stratifiers.

For peer review only - http://bmjopen.bmj.com/site/about/guidelines.xhtml

For the primary outcome, effect modification by (1) sex, (2) index of multiple deprivation (high, low), (3) educational qualification (below post-secondary, post-secondary and above), (4) duration of diabetes (<1 year; 1-3 years) will be tested using an F-test of the relevant multiplicative interaction parameter in the ANCOVA model. If the p-value for a particular interaction is <0.05, then the intervention effect and 95% CI will be estimated within the relevant subgroups.

Economic evaluation

A detailed health economics analysis plan will be developed and signed off by the Trial Steering Committee prior to analysis. An intention-to-treat within-trial cost effectiveness analysis will be conducted from both the NHS and the societal perspectives using participant-level data. Benefits will be measured by changes in HbA_{1c} at 12 months for the primary analysis and changes in weight (kg), HRQoL (EQ5D-5L), capability/wellbeing (ICECAP-A), and quality-adjusted life years at 12 months for the secondary analyses. Resource use data will be extracted from patient-completed questionnaires and validated with primary care notes review. Unit costs will be extracted from standard NHS cost databases and publications (e.g. NHS Reference Costs). Costs and benefits will be left undiscounted as follow up is only one-year. If required and appropriate, missing data will be imputed using recognised techniques such as multiple imputation. Descriptive statistics for resource use, total costs, HRQoL, and capability/wellbeing at 1 year will be reported as well as incremental cost-effectiveness and cost-utility ratios and incremental net (monetary) benefit measures. We will undertake deterministic and probabilistic sensitivity analyses, presenting the results of the latter as cost-effectiveness acceptability curves. Value of information analysis will quantify the value of reducing the decision uncertainty which will inform whether further research is worthwhile following completion of this study, and if so on which parameters.

Qualitative Evaluation

For peer review only

For peer review only - http://bmjopen.bmj.com/site/about/guidelines.xhtml

Qualitative analyses will explore how the programmes were implemented; participant and provider perceptions of the extent to which the programmes met patient needs; and factors participants and providers regarded as causally significant. Analyses will also explore the key challenges anticipated around treatment cessation and weight loss maintenance, and the value attached to the possibility of freedom from or reduction in medication and the concept of "remission".

Recordings will be transcribed by an experienced external agency and checked for accuracy by the research team. Verbatim transcripts will be coded using NVivo software, retaining a focus on narrative sequences and transitions as well as salient themes. A dual coding approach will be used: a first inductive round based on emerging themes relating to the research questions; a second round sensitised by quantitative findings. In the first inductive stage, open codes will be generated based on line-by-line scrutiny of verbatim transcripts uploaded into NVivo. Inconsistencies between coders will be resolved through discussion. Patient and Public Involvement (PPI) representatives will assist with the analysis of qualitative data; this will include coding a sub-sample of transcripts following training, and ensuing dialogue.

Trial Steering Committee

The Trial Steering Committee (TSC) will provide overall supervision for the GLoW Trial on behalf of the Trial Sponsors (NHS Cambridgeshire and Peterborough CCG, University of Cambridge) and Trial Funder (NIHR Clinical Commissioning Facility), and ensure that the project is conducted to the rigorous standards set out in the UK Policy Framework for Health and Social Care Research and the Guidelines for Good Clinical Practice. The TSC will provide advice to the Investigators on all aspects of the trial and will review and agree the trial protocol, the statistical analysis plan, and any amendments to the protocol. The TSC will be chaired by Professor Andrew Farmer (University of Oxford). Independent members include Professor Lucy Yardley (University of Southampton), Dr Thomas Fanshawe (University of Oxford), Dr Edel Doherty (NUI Galway), Mr Graham Rhodes (PPI

For peer review only - http://bmjopen.bmj.com/site/about/guidelines.xhtml

representative) and Ms Hazel Patel (PPI representative). This is a low risk trial in which participants in both trial groups are referred to accredited education programmes. There are no rules for early stopping and participants and GPs are not blind to intervention allocation. Thus a separate data monitoring committee is not deemed to be necessary.

Patient and Public Involvement

Patient and Public Involvement (PPI) informs the design, management, analysis and dissemination of the GLoW study. The initial ideas and research proposal were reviewed by 3 members of Fakenham Weight Management Service and 6 members of the University of Cambridge PPI Panel. A PPI representative (Jennifer Bostock) is a member of our Investigator team and has contributed to the design of the protocol. She will also contribute to designing and delivering PPI training, preparing ethics and R&D submissions, co-authoring journal articles and the final report, disseminating findings to a wide range of audiences, and supporting other PPI members. Two PPI representatives are members of the Trial Steering Committee. They will review the final study reports and contribute to the writing of specific sections, such as the lay summary.

To maximise participant engagement and retention, and minimise burden, PPI representatives review the content, design, and delivery of participant-facing materials. PPI representatives will also support the design of the qualitative interview schedule and the analysis and interpretation of qualitative data. Including PPI perspectives in plans for dissemination will ensure that we access an appropriate range of audiences and communicate messages effectively. PPI representatives will advise on content and methods of dissemination and will review public facing documents such as newsletters and press releases.

PPI representatives will be reimbursed for their time and expenses in a timely manner, and tailored PPI training will be provided to suit the specific needs of the individual and their role.

For peer review only - http://bmjopen.bmj.com/site/about/guidelines.xhtml

Ethics and Dissemination

The NHS Cambridgeshire and Peterborough CCG and University of Cambridge are the co-sponsors of the trial. The protocol and all participant facing materials were reviewed by the East of Scotland Research Ethics Service. Initial approval was received on 15th May 2018 (18/ES/0048) and HRA approval was granted on 31st May 2018. This protocol (version 3) was approved on 19th June 2019. If needed, the Investigators will submit any proposed substantial and non-substantial amendments to the protocol or other approved documents to the HRA for approval. Substantial amendments will not be implemented until relevant approvals have been given. The MRC Epidemiology Unit has an over-arching data management policy (DMP) that encompasses the standards and processes applied to all research and operational activities in the Unit. A study-specific data management plan, based on this DMP, details how data will be collected, stored, transferred, accessed and archived. The PIs will ensure that all data generated, stored and shared from this trial will be handled in compliance with the DMP and the General Data Protection Regulations.

All specified analyses will be written up as scientific papers and submitted for publication in peer-reviewed open-access journals. Members of the research team will be involved in reviewing drafts of the manuscripts, abstracts, and any other publications arising from the trial. The Principal Investigators will have final approval on all publications and any press release, where appropriate. Authorship will be determined using ICMJE criteria. Upon publication of the main findings, participants will be sent a newsletter that describes the results and gives details of who to contact to ask questions or obtain further information. Newsletters will be prepared with input from PPI representatives. Where appropriate, we will communicate our findings to local and national stakeholders via tailored summaries of the key findings, and by presentations at meetings of local and national networks. Representatives from these groups will be involved in our research throughout and will support us in identifying opportunities for dissemination.

Trial Status

For peer review only - http://bmjopen.bmj.com/site/about/guidelines.xhtml

This protocol (version 3) included additional strategies to boost recruitment and was approved on 19th June 2019. Recruitment for the trial began on 18th July 2018 and is expected to close in August 2020.

Author Contributions

ALA and SJG are joint principal investigators. ALA, SJG, SJS, RD, SM, AJH, BD, CAH, AB and JB are grant holders. JW and EWe are the trial coordinators. ALA, JW, EWe, SJS, NI, ERL, RD, AJH, BD, EWi, SM, CAH, AB, JB, CEB, SJG contributed to the design of the study protocol. ALA and JW wrote the first draft of the manuscript. ALA, JW, EWe, SJS, NI, ERL, RD, AJH, BD, EWi, SM, CAH, AB, JB, CEB, SJG contributed to the writing and critical revision of the manuscript.

Funding and Support in Kind

This study is funded by the National Institute for Health Research (NIHR) Programme Grants for Applied Research Programme (Reference Number RP-PG-0216-20010). The views expressed are those of the author(s) and not necessarily those of the NIHR or the Department of Health and Social Care. The tailored diabetes education and behavioural weight management programme (Live Well With Diabetes) is provided by WW (formerly Weight Watchers) free of charge for the purposes of this trial. ALA, SJS and SJG are supported by the Medical Research Council (grant MC_UU_12015/4). The University of Cambridge has received salary support in respect of SJG from the NHS in the East of England through the Clinical Academic Reserve. This is an investigator-led trial. None of the funders have any role in the study design, the analysis and interpretation of data, or the writing of the report and decision to submit the report for publication.

Competing Interests Statement

ALA is principal investigator on another publically funded trial where WW provided the intervention at no cost but has received no personal fees. SJG reports grants from Medical Research Council, personal fees from Eli Lilly, and personal fees from Janssen, outside this programme of research. AJH

For peer review only

For peer review only - http://bmjopen.bmj.com/site/about/guidelines.xhtml

reports personal fees from Slimming World and the College of Contemporary Health, outside this programme of research. CAH reports informal unpaid advice to Thriva. The other Investigators report no conflicts of interest.

Acknowledgements

We thank all staff from the MRC Epidemiology Unit Functional Group Team for input into the protocol design, particularly with regard to study coordination, anthropometry measurement, physical activity measurement, laboratory sample collection and analysis, data management, IT, business operations and research governance. We also thank NIHR CRN Eastern Primary Care research delivery colleagues, regional primary care stakeholders, and Cambridgeshire and Peterborough CCG Research Team for their input into the operational aspects of the study design and their comments on early iterations of the protocol.

Availability of data and materials

Following completion of the study and reporting of the main results, meta data will be made publically available via the lead institution's data sharing portal http://epi-meta.medschl.cam.ac.uk along with details of how to request data.

References

1. Hex N, Bartlett C, Wright D, Taylor M, Varley D. Estimating the current and future costs of Type1 and Type2 diabetes in the UK, including direct health costs and indirect societal and productivity costs. Diabetic Medicine. 2012;29(7):855–62.
2. HQIP, NHS, DUK. National Diabetes Audit , 2015-16 Report 2a : Complications and Mortality. 2017;(March).
3. Ali S, Stone MA, Peters JL, Davies MJ, Khunti K. The prevalence of co-morbid depression in adults with Type 2 diabetes: A systematic review and meta-analysis. Vol. 23, Diabetic Medicine.

For peer review only - http://bmjopen.bmj.com/site/about/guidelines.xhtml

2006. p. 1165–73.

4. Kanavos P, Aardweg S Van Den, Schurer W. Diabetes Expenditure, Burden of Disease and Management in 5 EU Countries. LSE Health [Internet]. 2012;(January):1–113. Available from: http://eprints.lse.ac.uk/54896/1/__libfile_REPOSITORY_Content_LSE Health and Social Care_Jan 2012_LSEDiabetesReport26Jan2012.pdf

5. Ribaric G, Buchwald JN, McGlennon TW. Diabetes and weight in comparative studies of bariatric surgery vs conventional medical therapy: A systematic review and meta-analysis. Obesity Surgery. 2014;24(3):437–55.

6. Sjöström L, Peltonen M, Jacobson P, Ahlin S, Andersson-Assarsson J, Anveden Å, et al. Association of bariatric surgery with long-term remission of type 2 diabetes and with microvascular and macrovascular complications. JAMA : the journal of the American Medical Association [Internet]. 2014;311(22):2297–304. Available from: http://www.ncbi.nlm.nih.gov/pubmed/24915261

7. Steven S, Hollingsworth K, Al-Mrabeh A, Avery L, Aribisala B, Caslake M, et al. Very-Low-Calorie Diet and 6 Months of Weight Stability in Type 2 Diabetes: Pathophysiologic Changes in Responders and Nonresponders. Diabetes care. 2016;March 21.

8. Lean MEJ, Leslie WS, Barnes AC, Brosnahan N, Thom G, McCombie L, et al. Primary care-led weight management for remission of type 2 diabetes (DiRECT): An open-label, cluster-randomised trial. The Lancet. 2017;

9. Gregg EW, Chen H, Wagenknecht LE, Clark JM, Delahanty LM, Bantle J, et al. Association of an intensive lifestyle intervention with remission of type 2 diabetes. JAMA : the journal of the American Medical Association [Internet]. 2012;308(23):2489–96. Available from: http://www.ncbi.nlm.nih.gov/pubmed/23288372

10. Long H G, Cooper AJM, Wareham J N, Griffin J S, Simmons K R. Healthy Behavior Change and Cardiovascular Outcomes in Newly Diagnosed Type 2 Diabetic Patients: A Cohort Analysis of the ADDITION-Cambridge Study. Diabetes Care [Internet]. 2014;37(6):1712–20. Available

For peer review only - http://bmjopen.bmj.com/site/about/guidelines.xhtml

from: http://search.ebscohost.com/login.aspx?direct=true&db=jlh&AN=2012593245&site=ehost-live

11. Strelitz J, Ahern AL, Long GH, Hare MJL, Irving G, Boothby CE, et al. Moderate weight change following diabetes diagnosis and 10 year incidence of cardiovascular disease and mortality. Diabetologia. 2019;

12. Strelitz J, Ahern AL, Long GH, Boothby CE, Wareham NJ, Griffin SJ. Changes in behaviors after diagnosis of type 2 diabetes and 10-year incidence of cardiovascular disease and mortality. Cardiovascular Diabetology. 2019;

13. Dambha-Miller H, Day AJ, Strelitz J, Irving G, Griffin SJ. Behaviour change, weight loss and remission of Type 2 diabetes: a community-based prospective cohort study. Diabetic Medicine. 2019;

14. Wadden TA. Eight-year weight losses with an intensive lifestyle intervention: The look AHEAD study. Obesity. 2014;22:5–13.

15. NICE NG28. Type 2 diabetes in adults: management. National Institute for Health and Care Excellence [Internet]. 2015;(December 2015):1–86. Available from: http://www.nice.org.uk/guidance/ng28/resources/type-2-diabetes-in-adults-management-1837338615493

16. Davies MJ, Heller S, Skinner TC, Campbell MJ, Carey ME, Cradock S, et al. Effectiveness of the diabetes education and self management for ongoing and newly diagnosed (DESMOND) programme for people with newly diagnosed type 2 diabetes: cluster randomised controlled trial. BMJ (Clinical research ed) [Internet]. 2008;336(7642):491–5. Available from: http://www.pubmedcentral.nih.gov/articlerender.fcgi?artid=2258400&tool=pmcentrez&rendertype=abstract

17. Pillay J, Armstrong MJ, Butalia S, Donovan LE, Sigal RJ, Vandermeer B, et al. Behavioral Programs for Type 2 Diabetes Mellitus: A Systematic Review and Network Meta-analysis. Annals of internal medicine [Internet]. 2015 Dec 1;163(11):848–60. Available from:

For peer review only

For peer review only - http://bmjopen.bmj.com/site/about/guidelines.xhtml

http://annals.org/article.aspx?articleid=2446188%5Cnhttp://www.ncbi.nlm.nih.gov/pubmed/26414227

18. Gillett M, Dallosso HM, Dixon S, Brennan A, Carey ME, Campbell MJ, et al. Delivering the diabetes education and self management for ongoing and newly diagnosed (DESMOND) programme for people with newly diagnosed type 2 diabetes: cost effectiveness analysis. BMJ (Clinical research ed) [Internet]. 2010;341:c4093. Available from: http://www.pubmedcentral.nih.gov/articlerender.fcgi?artid=2924963&tool=pmcentrez&rendertype=abstract

19. Ahern AL, Wheeler GM, Aveyard P, Boyland EJ, Halford JCG, Mander AP, et al. Extended and standard duration weight-loss programme referrals for adults in primary care (WRAP): a randomised controlled trial. The Lancet. 2017;389(10085):2214–25.

20. Meads DM, Hulme CT, Hall P, Hill AJ. The cost-effectiveness of primary care referral to a UK commercial weight loss programme. Clinical obesity. 2014;4(6):324–32.

21. Fuller NR, Colagiuri S, Schofield D, Olson AD, Shrestha R, Holzapfel C, et al. A within-trial cost-effectiveness analysis of primary care referral to a commercial provider for weight loss treatment, relative to standard care--an international randomised controlled trial. International Journal of Obesity. England; 2013 Jun;37(6):828–34.

22. Jebb SA, Ahern AL, Olson AD, Aston LM, Holzapfel C, Stoll J, et al. Primary care referral to a commercial provider for weight loss treatment versus standard care: a randomised controlled trial. The Lancet [Internet]. 2011;378(9801):1485–92. Available from: http://www.ncbi.nlm.nih.gov/pubmed/21906798

23. O'Neil PM, Miller-Kovach K, Tuerk PW, Becker LE, Wadden TA, Fujioka K, et al. Randomized controlled trial of a nationally available weight control program tailored for adults with type 2 diabetes. Obesity. 2016;24(11):2269–77.

24. Herdman M, Gudex C, Lloyd A, Janssen M, Kind P, Parkin D, et al. Development and preliminary testing of the new five-level version of EQ-5D (EQ-5D-5L). Quality of Life Research.

For peer review only - http://bmjopen.bmj.com/site/about/guidelines.xhtml

2011;20(10):1727–36.

25. Devlin NJ, Shah KK, Feng Y, Mulhern B, van Hout B. Valuing health-related quality of life: An EQ-5D-5L value set for England. Health Economics (United Kingdom). 2018;27(1):7–22.

26. Al-Janabi H, Flynn TN, Coast J. Development of a self-report measure of capability wellbeing for adults: The ICECAP-A. Quality of Life Research. 2012;21(1):167–76.

27. Flynn TN, Huynh E, Peters TJ, Al-Janabi H, Clemens S, Moody A, et al. Scoring the ICECAP-A capability instrument. Estimation of a UK general population tariff. Health Economics (United Kingdom). 2015;24(3):258–69.

28. Doherty A, Jackson D, Hammerla N, Plötz T, Olivier P, Granat MH, et al. Large scale population assessment of physical activity using wrist worn accelerometers: The UK biobank study. PLoS ONE. 2017;12(2).

29. Wareham NJ, Jakes RW, Rennie KL, Mitchell J, Hennings S, Day NE. Validity and repeatability of the EPIC-Norfolk Physical Activity Questionnaire. International journal of epidemiology. 2002;31(1):168–74.

30. Golubic R, May AM, Benjaminsen Borch K, Overvad K, Charles MA, Diaz MJT, et al. Validity of electronically administered Recent Physical Activity Questionnaire (RPAQ) in ten European countries. PLoS ONE. 2014;9(3).

31. Mulligan AA, Luben RN, Bhaniani A, Parry-Smith DJ, O'Connor L, Khawaja AP, et al. A new tool for converting food frequency questionnaire data into nutrient and food group values: FETA research methods and availability. BMJ Open. 2014;4(3).

32. O'Neill J, Tabish H, Welch V, Petticrew M, Pottie K, Clarke M, et al. Applying an equity lens to interventions: Using PROGRESS ensures consideration of socially stratifying factors to illuminate inequities in health. Journal of Clinical Epidemiology. 2014;67(1):56–64.

33. Bradley C, Todd C, Gorton T, Symonds E, Martin A, Plowright R. The development of an individualized questionnaire measure of perceived impact of diabetes on quality of life: The ADDQoL. Quality of Life Research. 1999;8(1–2):79–91.

For peer review only

For peer review only - http://bmjopen.bmj.com/site/about/guidelines.xhtml

34. Dalton M, Finlayson G, Hill A, Blundell J. Preliminary validation and principal components analysis of the Control of Eating Questionnaire (CoEQ) for the experience of food craving. European Journal of Clinical Nutrition. 2015;69(12):1313–7.

35. Westenhoefer J, Stunkard AJ, Pudel V. Validation of the flexible and rigid control dimensions of dietary restraint. International Journal of Eating Disorders. 1999;26:53–64.

36. Gormally J, Black S, Daston S, Rardin D. The assessment of binge eating severity among obese persons. Addictive Behaviors. 1982;7(1):47–55.

37. Grupski AE, Hood MM, Hall BJ, Azarbad L, Fitzpatrick SL, Corsica JA. Examining the binge eating scale in screening for binge eating disorder in bariatric surgery candidates. Obesity Surgery. 2013;23(1):1–6.

38. Griffin SJ, Borch-Johnsen K, Davies MJ, Khunti K, Rutten GE, Sandbæk A, et al. Effect of early intensive multifactorial therapy on 5-year cardiovascular outcomes in individuals with type 2 diabetes detected by screening (ADDITION-Europe): a cluster-randomised trial. The Lancet [Internet]. 2011 Jul;378(9786):156–67. Available from: http://linkinghub.elsevier.com/retrieve/pii/S0140673611606983

39. White IR, Thompson SG. Adjusting for partially missing baseline measurements in randomized trials. Statistics in Medicine. 2005;

For peer review only

For peer review only - http://bmjopen.bmj.com/site/about/guidelines.xhtml

Figure 1: Participant Flow Diagram

Figure 2: Schedule of Enrolment, Interventions and Assessments

For peer review only

For peer review only - http://bmjopen.bmj.com/site/about/guidelines.xhtml

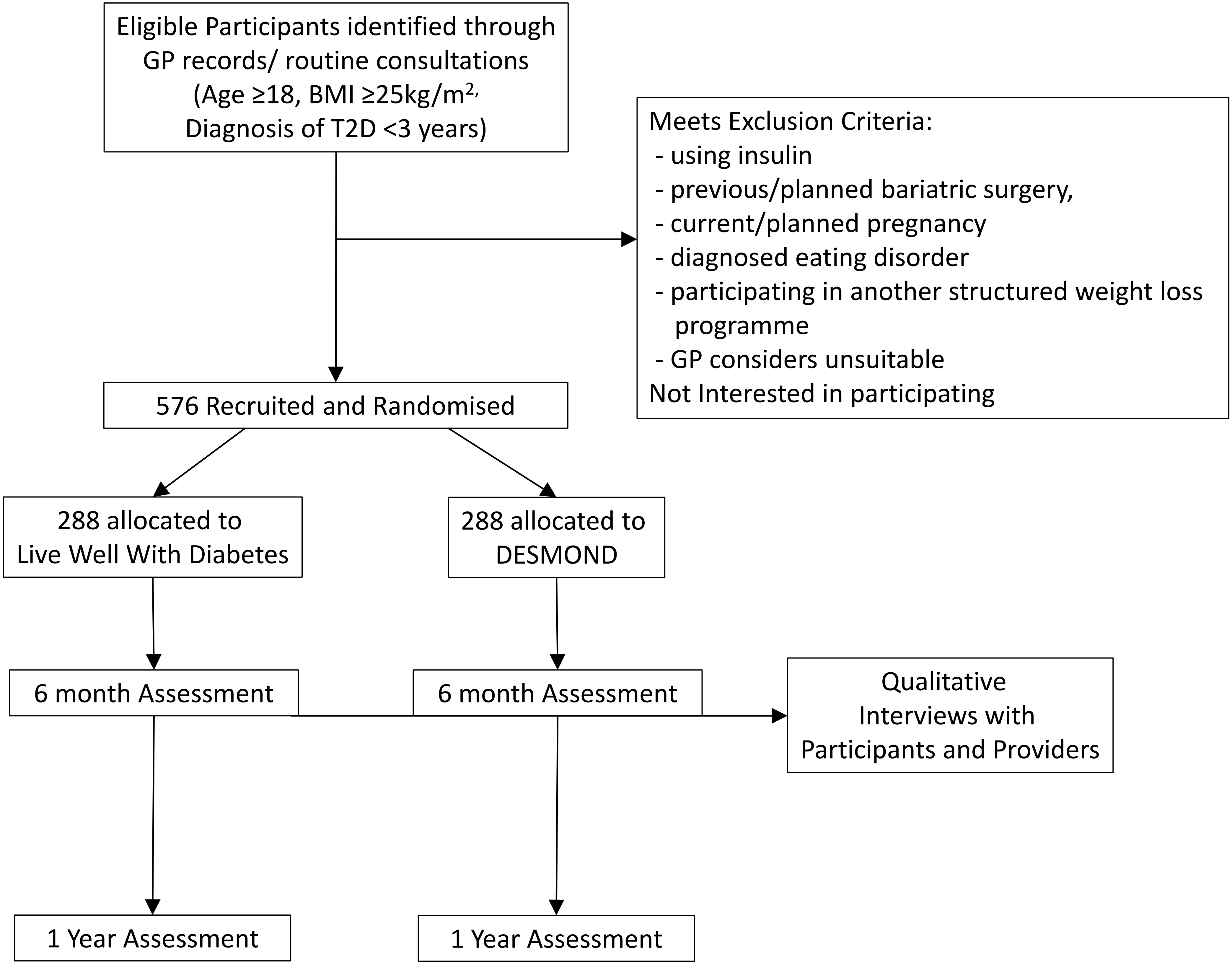
Eligible Participants identified through GP records/ routine consultations (Age ≥18, BMI ≥25kg/m2, Diagnosis of T2D <3 years)
Meets Exclusion Criteria:
- using insulin
- previous/planned bariatric surgery,
- current/planned pregnancy
- diagnosed eating disorder
- participating in another structured weight loss programme
- GP considers unsuitable
Not Interested in participating
576 Recruited and Randomised
288 allocated to Live Well With Diabetes
288 allocated to DESMOND
6 month Assessment
6 month Assessment
Qualitative Interviews with Participants and Providers
1 Year Assessment
1 Year Assessment

	STUDY PERIOD			
	**Enrolment**	**Baseline**	**Follow Up**	
**TIMEPOINT****	***-t_1***	**0**	***6 months***	***12 months***
**ENROLMENT:**				
**Telephone Eligibility screen**	X			
**Informed consent**		X		
**Randomisation**		X		
**INTERVENTIONS:**				
Tailored diabetes education and behavioural weight management		◆——	——◆	
Diabetes Education		X		
**ASSESSMENTS:**				
**Height**		X		
**Weight**		X	X	X
**Body fat %**		X	X	X
**Blood Pressure**		X	X	X
**Blood sample**		X	X	X
**Accelerometer**		X	X	X
**Diet Recall**		X	X	X
**Self-Report Questionnaires**		X	X	X
**Notes Review**		X	X	X
**Registry Data**				X

Glucose Lowering through Weight management (GLoW)

Participant study number:

Barcode

CONSENT FORM

Version 2.0 (16/01/2019)

REC Ref:18/ES/0048 IRAS ID:236767 Chief Investigator: Dr Amy Ahern

For peer review only

Please initial each box

1. I confirm that I have read and understood the information sheet **version 2.0, 04/12/2018** for the above study and have had the opportunity to consider the information, ask questions and have had these answered satisfactorily.
2. I understand that my participation is voluntary and that I am free to withdraw at any time, without giving any reason and without my medical care being negatively affected or my legal rights.
3. I understand that samples I have given and the information collected about me can be stored by the MRC Epidemiology Unit, University of Cambridge.
4. I agree to give a blood sample for this research study.
5. I give permission that my samples may be analysed in an accredited laboratory outside of the MRC Epidemiology Unit, University of Cambridge. All samples will be made anonymous and no personal identifiable information will be sent to another laboratory.
6. I understand that in order to conduct this study, the research team, my GP, and the intervention providers may need to share personal information about me, such as my contact information and details of my health status (e.g. diabetes status), with each other. I understand that this data will only be used for research purposes or to ensure I receive the best clinical care and I give permission for my personal information to be used in this way.
7. I understand that sections of my medical notes may be looked at by members of the research team, regulatory authorities or by the NHS Trust**,** where it is relevant to my taking part in this research study. I give permission for these individuals to have access to my records, and for this data to be linked to my study data.
8. I understand that I will not benefit financially if this research leads to the development of a new treatment or test.
9. I agree to take part in the above study

Optional

Yes No

10. I agree that my GP will be notified of my participation in this research and I agree to my GP being forwarded my results.

White copy for the researcher, yellow for the GP, pink for the participant

For peer review only - http://bmjopen.bmj.com/site/about/guidelines.xhtml

Barcode

	Yes	No
11. I understand that my anonymised samples and data will be used to support other research in the future, and may be shared anonymously with other researchers. This could include collaborators overseas or in the commercial sector.	☐	☐
12. I give permission for future medical notes reviews to be conducted up to 15 years following my participation in this study, in order to collect long term information about my health.	☐	☐
13. I give permission for the research team to obtain personal and health-related data from NHS Digital and from national health and social care registries for up to 15 years following the end of the study and to add this information to my study data. I understand that to do this, the research team may need to give a small amount of personal information about me (e.g. name, date of birth, address, GP) to these organisations. I consent to my study data being linked to:	☐	☐

- Myocardial Ischaemia National Audit Project (MINAP) ☐
- Sentinel Stroke National Audit Programme (SSNAP) ☐
- Cancer registry ☐
- Office of National Statistics (ONS) ☐
- Civil Registration Mortality Data ☐
- Hospital Episode Statistics (HES) ☐

	Yes	No
14. I am willing to be contacted again in the future beyond this study and any follow up from it.	☐	☐
15. In the event that my contact details change, I am willing for the research team to attempt to find out my new contact details through my GP, the postal service, or NHS Digital.	☐	☐

-- Full name of Participant (BLOCK CAPITALS)	☐☐ ☐☐ ☐☐☐☐ Date (DD MM YYYY)	--------------------- Signature
-- Full name of Researcher	☐☐ ☐☐ ☐☐☐☐ Date (DD MM YYYY)	--------------------- Signature

REC: 18/ES/0048
IRAS ID: 236767 16/01/2019 (version 2.0)

For peer review only - http://bmjopen.bmj.com/site/about/guidelines.xhtml

SPIRIT

STANDARD PROTOCOL ITEMS: RECOMMENDATIONS FOR INTERVENTIONAL TRIALS

SPIRIT 2013 Checklist: Recommended items to address in a clinical trial protocol and related documents*

Section/item	Item No	Description	
**Administrative information**			
Title	1	Descriptive title identifying the study design, population, interventions, and, if applicable, trial acronym	1
Trial registration	2a	Trial identifier and registry name. If not yet registered, name of intended registry	3
	2b	All items from the World Health Organization Trial Registration Data Set	Throughout
Protocol version	3	Date and version identifier	2
Funding	4	Sources and types of financial, material, and other support	25
Roles and responsibilities	5a	Names, affiliations, and roles of protocol contributors	24
	5b	Name and contact information for the trial sponsor	ISRCTN
	5c	Role of study sponsor and funders, if any, in study design; collection, management, analysis, and interpretation of data; writing of the report; and the decision to submit the report for publication, including whether they will have ultimate authority over any of these activities	25
	5d	Composition, roles, and responsibilities of the coordinating centre, steering committee, endpoint adjudication committee, data management team, and other individuals or groups overseeing the trial, if applicable (see Item 21a for data monitoring committee)	22
**Introduction**			
Background and rationale	6a	Description of research question and justification for undertaking the trial, including summary of relevant studies (published and unpublished) examining benefits and harms for each intervention	4
	6b	Explanation for choice of comparators	4
Objectives	7	Specific objectives or hypotheses	5
Trial design	8	Description of trial design including type of trial (eg, parallel group, crossover, factorial, single group), allocation ratio, and framework (eg, superiority, equivalence, noninferiority, exploratory)	6

For peer review only

Methods: Participants, interventions, and outcomes

Study setting	9	Description of study settings (eg, community clinic, academic hospital) and list of countries where data will be collected. Reference to where list of study sites can be obtained	7
Eligibility criteria	10	Inclusion and exclusion criteria for participants. If applicable, eligibility criteria for study centres and individuals who will perform the interventions (eg, surgeons, psychotherapists)	7
Interventions	11a	Interventions for each group with sufficient detail to allow replication, including how and when they will be administered	8
	11b	Criteria for discontinuing or modifying allocated interventions for a given trial participant (eg, drug dose change in response to harms, participant request, or improving/worsening disease)	n/a
	11c	Strategies to improve adherence to intervention protocols, and any procedures for monitoring adherence (eg, drug tablet return, laboratory tests)	15
Outcomes	12	Primary, secondary, and other outcomes, including the specific measurement variable (eg, systolic blood pressure), analysis metric (eg, change from baseline, final value, time to event), method of aggregation (eg, median, proportion), and time point for each outcome. Explanation of the clinical relevance of chosen efficacy and harm outcomes is strongly recommended	10
Participant timeline	13	Time schedule of enrolment, interventions (including any run-ins and washouts), assessments, and visits for participants. A schematic diagram is highly recommended (see Figure)	Figure 2
Sample size	14	Estimated number of participants needed to achieve study objectives and how it was determined, including clinical and statistical assumptions supporting any sample size calculations	20
Recruitment	15	Strategies for achieving adequate participant enrolment to reach target sample size	17

Methods: Assignment of interventions (for controlled trials)

Allocation:

Sequence generation	16a	Method of generating the allocation sequence (eg, computer-generated random numbers), and list of any factors for stratification. To reduce predictability of a random sequence, details of any planned restriction (eg, blocking) should be provided in a separate document that is unavailable to those who enrol participants or assign interventions	19

For peer review only - http://bmjopen.bmj.com/site/about/guidelines.xhtml

Allocation concealment mechanism	16b	Mechanism of implementing the allocation sequence (eg, central telephone; sequentially numbered, opaque, sealed envelopes), describing any steps to conceal the sequence until interventions are assigned	19
Implementation	16c	Who will generate the allocation sequence, who will enrol participants, and who will assign participants to interventions	19
Blinding (masking)	17a	Who will be blinded after assignment to interventions (eg, trial participants, care providers, outcome assessors, data analysts), and how	19
	17b	If blinded, circumstances under which unblinding is permissible, and procedure for revealing a participant's allocated intervention during the trial	19
**Methods: Data collection, management, and analysis**			
Data collection methods	18a	Plans for assessment and collection of outcome, baseline, and other trial data, including any related processes to promote data quality (eg, duplicate measurements, training of assessors) and a description of study instruments (eg, questionnaires, laboratory tests) along with their reliability and validity, if known. Reference to where data collection forms can be found, if not in the protocol	12
	18b	Plans to promote participant retention and complete follow-up, including list of any outcome data to be collected for participants who discontinue or deviate from intervention protocols	12
Data management	19	Plans for data entry, coding, security, and storage, including any related processes to promote data quality (eg, double data entry; range checks for data values). Reference to where details of data management procedures can be found, if not in the protocol	24
Statistical methods	20a	Statistical methods for analysing primary and secondary outcomes. Reference to where other details of the statistical analysis plan can be found, if not in the protocol	20
	20b	Methods for any additional analyses (eg, subgroup and adjusted analyses)	20-22
	20c	Definition of analysis population relating to protocol non-adherence (eg, as randomised analysis), and any statistical methods to handle missing data (eg, multiple imputation)	20
**Methods: Monitoring**			
Data monitoring	21a	Composition of data monitoring committee (DMC); summary of its role and reporting structure; statement of whether it is independent from the sponsor and competing interests; and reference to where further details about its charter can be found, if not in the protocol. Alternatively, an explanation of why a DMC is not needed	22

	21b	Description of any interim analyses and stopping guidelines, including who will have access to these interim results and make the final decision to terminate the trial	23
Harms	22	Plans for collecting, assessing, reporting, and managing solicited and spontaneously reported adverse events and other unintended effects of trial interventions or trial conduct	23
Auditing	23	Frequency and procedures for auditing trial conduct, if any, and whether the process will be independent from investigators and the sponsor	23
**Ethics and dissemination**			
Research ethics approval	24	Plans for seeking research ethics committee/institutional review board (REC/IRB) approval	24
Protocol amendments	25	Plans for communicating important protocol modifications (eg, changes to eligibility criteria, outcomes, analyses) to relevant parties (eg, investigators, REC/IRBs, trial participants, trial registries, journals, regulators)	24
Consent or assent	26a	Who will obtain informed consent or assent from potential trial participants or authorised surrogates, and how (see Item 32)	18
	26b	Additional consent provisions for collection and use of participant data and biological specimens in ancillary studies, if applicable	n/a
Confidentiality	27	How personal information about potential and enrolled participants will be collected, shared, and maintained in order to protect confidentiality before, during, and after the trial	24
Declaration of interests	28	Financial and other competing interests for principal investigators for the overall trial and each study site	25
Access to data	29	Statement of who will have access to the final trial dataset, and disclosure of contractual agreements that limit such access for investigators	26
Ancillary and post-trial care	30	Provisions, if any, for ancillary and post-trial care, and for compensation to those who suffer harm from trial participation	n/a
Dissemination policy	31a	Plans for investigators and sponsor to communicate trial results to participants, healthcare professionals, the public, and other relevant groups (eg, via publication, reporting in results databases, or other data sharing arrangements), including any publication restrictions	24
	31b	Authorship eligibility guidelines and any intended use of professional writers	24
	31c	Plans, if any, for granting public access to the full protocol, participant-level dataset, and statistical code	26

For peer review only - http://bmjopen.bmj.com/site/about/guidelines.xhtml

Appendices

Informed consent materials	32	Model consent form and other related documentation given to participants and authorised surrogates	Supplied to HRA
Biological specimens	33	Plans for collection, laboratory evaluation, and storage of biological specimens for genetic or molecular analysis in the current trial and for future use in ancillary studies, if applicable	Supplied to HRA

*It is strongly recommended that this checklist be read in conjunction with the SPIRIT 2013 Explanation & Elaboration for important clarification on the items. Amendments to the protocol should be tracked and dated. The SPIRIT checklist is copyrighted by the SPIRIT Group under the Creative Commons "Attribution-NonCommercial-NoDerivs 3.0 Unported" license.

For peer review only
