# Supplementary material for: Clinical and cost-effectiveness of a diabetes education and behavioural weight management programme versus a diabetes education programme in adults with a recent diagnosis of type 2 diabetes: study protocol for the Glucose Lowering through Weight management (GLoW) randomised controlled trial
Source: BMJ Open. 2020 Apr 28;10(4):e035020. doi: 10.1136/bmjopen-2019-035020 (PMC7213851; doi:10.1136/bmjopen-2019-035020)
Supplement: Reviewer comments [file bmjopen-2019-035020.reviewer_comments.pdf]

PEER REVIEW HISTORY

BMJ Open publishes all reviews undertaken for accepted manuscripts. Reviewers are asked to complete a checklist review form (**http://bmjopen.bmj.com/site/about/resources/checklist.pdf**) and are provided with free text boxes to elaborate on their assessment. These free text comments are reproduced below.

ARTICLE DETAILS

**TITLE (PROVISIONAL)**	The clinical and cost-effectiveness of a diabetes education and behavioural weight management programme versus a diabetes education programme in adults with a recent diagnosis of type 2 diabetes: Study protocol for the Glucose Lowering through Weight Management (GLoW) randomised controlled trial.
**AUTHORS**	Ahern, Amy L.; Woolston, Jenny; Wells, Emma; Sharp, Stephen; Islam, Nazrul; Lawlor, Emma; Duschinsky, Robbie; Hill, Andrew; Doble, Brett; Wilson, Ed; Morris, Stephen; Hughes, Carly; Brennan, Alan; Bostock, Jennifer; Boothby, Clare; Griffin, Simon J

This article was not externally reviewed at BMJ Open. Protocols that have been independently assessed prior to submission to BMJ Open are usually fast-tracked to publication on the grounds that further substantial changes will not be possible. This independent assessment will usually be external, independent review for both a substantial grant award from a non-commercial or government funder and ethics approval.
